# Identification of an *I*_Na_-dependent and *I*_to_-mediated proarrhythmic mechanism in cardiomyocytes derived from pluripotent stem cells of a Brugada syndrome patient

**DOI:** 10.1038/s41598-018-29574-5

**Published:** 2018-07-26

**Authors:** Dongrui Ma, Zhenfeng Liu, Li Jun Loh, Yongxing Zhao, Guang Li, Reginald Liew, Omedul Islam, Jianjun Wu, Ying Ying Chung, Wee Siong Teo, Chi Keong Ching, Boon Yew Tan, Daniel Chong, Kah Leng Ho, Paul Lim, Rita Yu Yin Yong, Brian K. Panama, Aaron D. Kaplan, Glenna C. L. Bett, James Ware, Connie R. Bezzina, Arie O. Verkerk, Stuart A. Cook, Randall L. Rasmusson, Heming Wei

**Affiliations:** 1 National Heart Research Institute Singapore, National Heart Centre Singapore, Singapore, 169609 Republic of Singapore; 2 Cardiovascular & Metabolic Disorders Program, Duke-NUS Medical School Singapore, Singapore, 169857 Republic of Singapore; 3 Department of Cardiology, National Heart Centre Singapore, Singapore, 169609 Republic of Singapore; 4 Defense Medical and Environmental Research Institute, DSO National Laboratories, Singapore, 117510 Republic of Singapore; 5 University at Buffalo, State University of New York, Buffalo, NY 14214 USA; 6 Imperial College London, South Kensington Campus, London, SW7 2AZ UK; 7 Academic Medical Center, University of Amsterdam, Amsterdam, The Netherlands

## Abstract

Brugada syndrome (BrS) is an inherited cardiac arrhythmia commonly associated with SCN5A mutations, yet its ionic mechanisms remain unclear due to a lack of cellular models. Here, we used human induced pluripotent stem cell-derived cardiomyocytes (hiPSC-CMs) from a BrS patient (BrS1) to evaluate the roles of Na^+^ currents (*I*_Na_) and transient outward K^+^ currents (*I*_to_) in BrS induced action potential (AP) changes. To understand the role of these current changes in repolarization we employed dynamic clamp to “electronically express” *I*_K1_ and restore normal resting membrane potentials and allow normal recovery of the inactivating currents, *I*_Na_, *I*_Ca_ and *I*_to_. HiPSC-CMs were generated from BrS1 with a compound SCN5A mutation (*p*. A226V & *p*. R1629X) and a healthy sibling control (CON1). Genome edited hiPSC-CMs (BrS2) with a milder *p*. T1620M mutation and a commercial control (CON2) were also studied. CON1, CON2 and BrS2, had unaltered peak *I*_Na_ amplitudes, and normal APs whereas BrS1, with over 75% loss of *I*_Na_, displayed a loss-of-*I*_Na_ basal AP morphology (at 1.0 Hz) manifested by a reduced maximum upstroke velocity (by ~80%, p < 0.001) and AP amplitude (p < 0.001), and an increased phase-1 repolarization pro-arrhythmic AP morphology (at 0.1 Hz) in ~25% of cells characterized by marked APD shortening (~65% shortening, p < 0.001). Moreover, *I*_to_ densities of BrS1 and CON1 were comparable and increased from 1.0 Hz to 0.1 Hz by ~ 100%. These data indicate that a repolarization deficit could be a mechanism underlying BrS.

## Introduction

The Brugada syndrome (BrS) is a rare cardiac rhythm disorder associated with an increased risk of malignant ventricular arrhythmias^1,2^. The signature ‘coved-type’ ST-segment elevation in the right precordial leads (V1–V3) of the electrocardiogram (ECG) may occur spontaneously or be induced by a provocative drug test with sodium channel blocking drugs^1,2^. Fetal arrhythmic events in BrS often occur during sleep/rest, a condition associated with slow heart rates^3^.

*SCN5A* encodes the pore-forming subunit (Na_v_1.5) of the cardiac sodium channels. *SCN5A* variants account for ~80% of BrS, with known genetic mutations^4^, and there are over 300 genetic variants in *SCN5A* associated with BrS^4,5^. Several studies have established loss of sodium channel function associated with these *SCN5A* variants^5,6^. However, the low penetrance and variable expressivity of *SCN5A* reported for BrS raise questions about the quantitative role of the Na^+^ current in the ionic mechanism of BrS.

A *repolarization disorder hypothesis* of BrS has been made based on studies using perfused ventricular wedges and isolated cardiac myocytes of animals^7,8^. Yet the role of the decreased Na_v_1.5 current (*I*_Na_) remains to be defined. The genetically engineered haploinsufficient (*SCN5A*^+/−^)^9^ and *SCN5A*^1798insD/+^^10^ mouse models, and the *SCN5A*^E558X/+^ pig model^11^, have shown mainly conduction defects without the *loss-of-dome* like *increased phase-1 repolarization* change in the action potential (AP). Moreover, from the two latest reports in which BrS patient-specific human induced pluripotent stem cells (hiPSC) derived cardiomyocytes (hiPSC-CMs) were adopted, no *loss-of-dome* like AP change was observed^12,13^.

In the current study, we selected hiPSC-CMs generated from a BrS patient with a severe reduction in *I*_Na_^14^. Next, we converted the hiPSC-CMs to more mature, native ventricular myocyte-like cells by *in silico* injection of a synthetic inward rectifier K^+^ current (*I*_K1_)^15^ and challenged the cells with slow pacing frequencies. We then identified an *I*_Na_ deficiency-dependent *loss-of-I*_Na_ basal AP pattern of BrS at normal heart rates and an *increased phase-1 repolarization* proarrhythmic AP pattern at a low heart rate. We further obtained evidence that associated the heart rate-induced AP changes with transient outward K^+^ current (*I*_to_). We therefore conclude that a *loss-of-I*_*Na*_ and an elevated *I*_to_ could together make the ventricular BrS cardiomyocytes undergo proarrhythmic changes.

## Results

### Impact of *SCN5A* mutations of BrS1 on *SCN5A*/Na_v_1.5 expression and *I*_Na_

**Figure 1:**
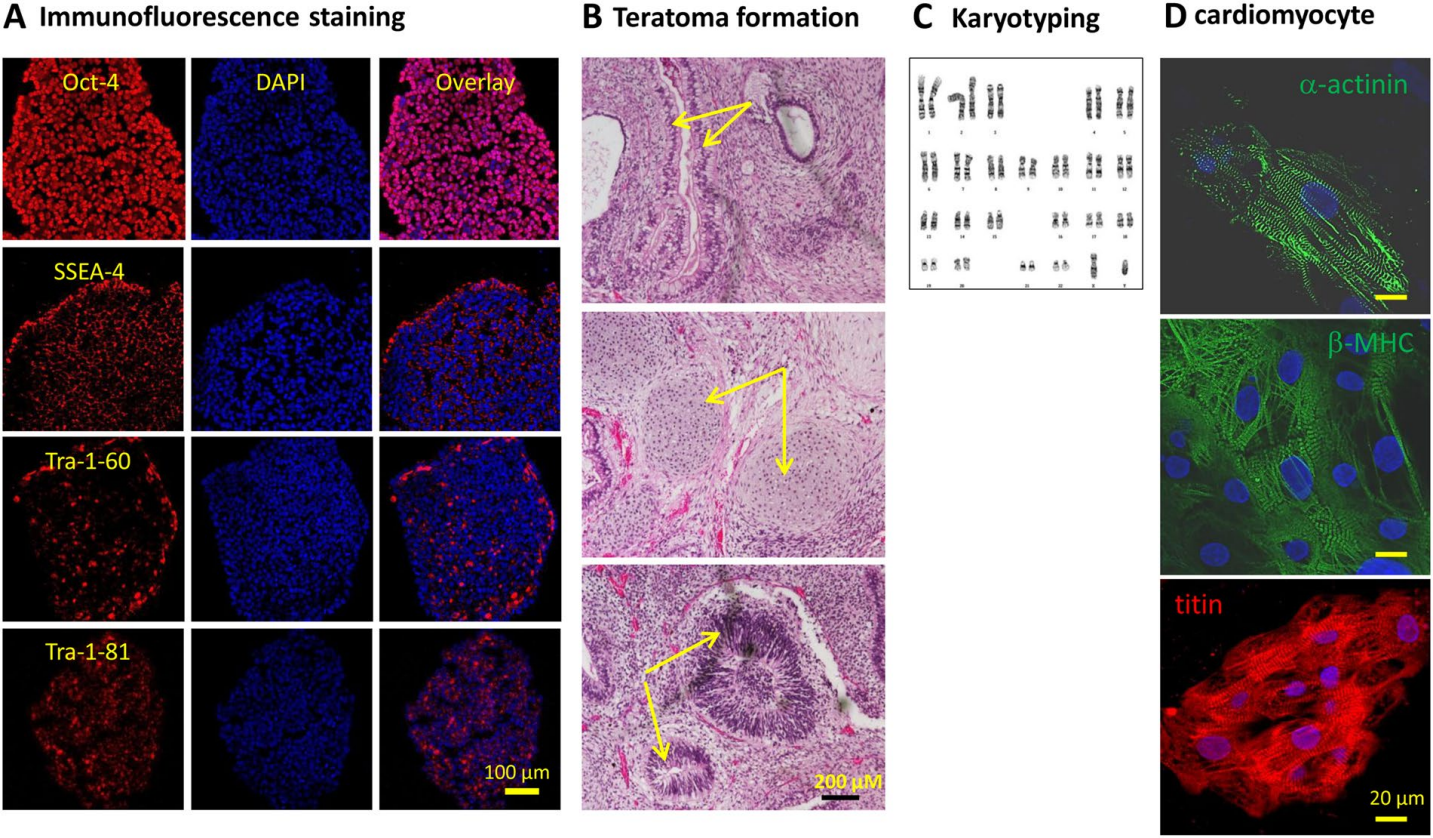
Characterization of hiPSC line and hiPSC-CMs. Representative data from BrS hiPSCs are presented. (**A**) The expression of pluripotent stem cell markers Oct-4, SSEA-4, Tra-1–60 and Tra-1–81; (**B**) Teratoma formation in SCID mice after hiPSCs injection confirmed by identifying three primitive germ layers: intestine (top) for endoderm, cartilage (middle) for mesoderm and neuroepithelium (bottom) for ectoderm. (**C**) Normal karyotype identified with BrS hiPSCs. (**D**) markers of cardiomyocytes identified with hiPSC-CMs. Top: α-actinin; Middle: β-MHC; and Bottom: cardiac titin.

**Figure 2:**
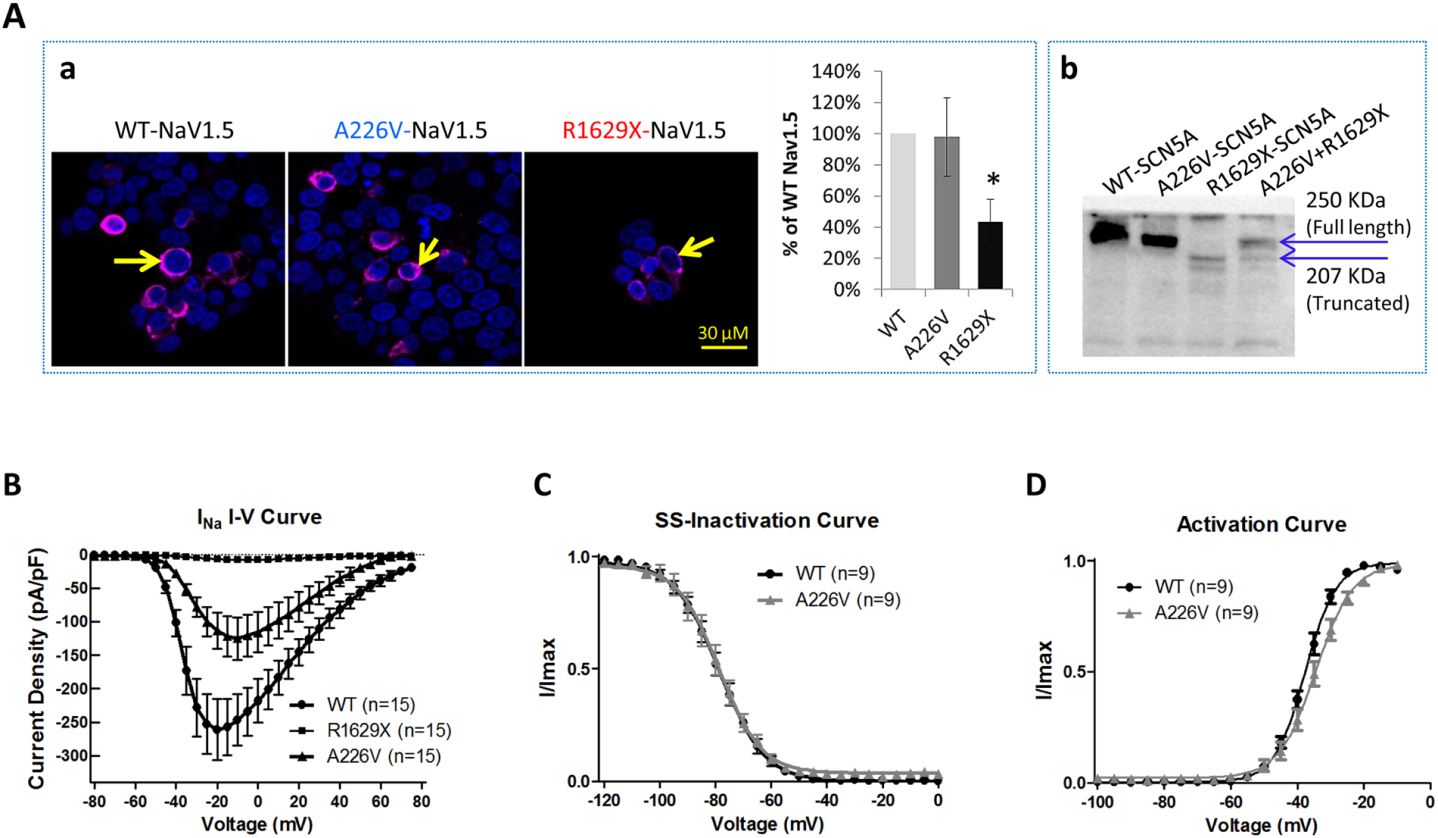
Expression of *SCN5A*/Nav1.5 and *I*_Na_ density documented in tsa201 heterologous expression system. tsa201 cells transfected with equal amount of WT-SCN5A, A226V-SCN5A, R1629X-*SCN5A* and A226V-*SCN5A* + R1629X-*SCN5A* (50% each) were assayed for *SCN5A*/Nav1.5 expression and *I*_Na_ density. (**Aa**) Representative immunofluorescence staining images of Na_v_1.5 (purple) in tsa201cells. Arrows indicate the Na_v_1.5 staining. Levels of Na_v_1.5 were semi-quantified and plotted in a bar-graph (n = 3). (**Ab**) Representative Western blotting image of Na_v_1.5 in tsa201 transfected cells (n = 3). (**B**) Peak *I*_Na_ density measured in transfected tsa201 cells. (**C**) and (**D**) Steady state (SS)-inactivation and activation curves of *I*_Na_ measured in tsa201 expressing WT- and A226V-SCN5A. *p < 0.05; vs. WT-*SCN5A*.

A BrS patient with *p*.A226V and *p*.R1629X mutations of *SCN5A* was selected in this study (Fig. 1). First, Tsa201 cells heterologously expressing WT-, A226V- and R1629X-*SCN5A* were prepared. Immunofluorescence (Fig. 2Aa) and Western blotting (Fig. 2Ab) assays showed that, compared with WT control, A226V-*SCN5A* cells expressed comparable levels of full-length Na_v_1.5, whereas R1629X-*SCN5A* cells showed a much lower level and smaller size of a truncated Na_v_1.5. Cells co-expressing A226V-*SCN5A* and R1629X-*SCN5A* showed both the full length and truncated Na_v_1.5. Next, *I*_Na_ was recorded in tsa201 cells heterologously expressing WT-, A226V- and R1629X-*SCN5A*. The average *I*_Na_ density (pA/pF) in A226V-*SCN5A* cells was ~50% of WT-*SCN5A* (*p* < 0.01) and it was almost undetectable in R1629X-*SCN5A* cells (Table S1, Fig. 2B). The steady state (SS)-inactivation (Fig. 2C) and -activation (Fig. 2D) of *I*_Na_ in A226V-*SCN5A* cells were largely unaffected (Table S1).

**Figure 3:**
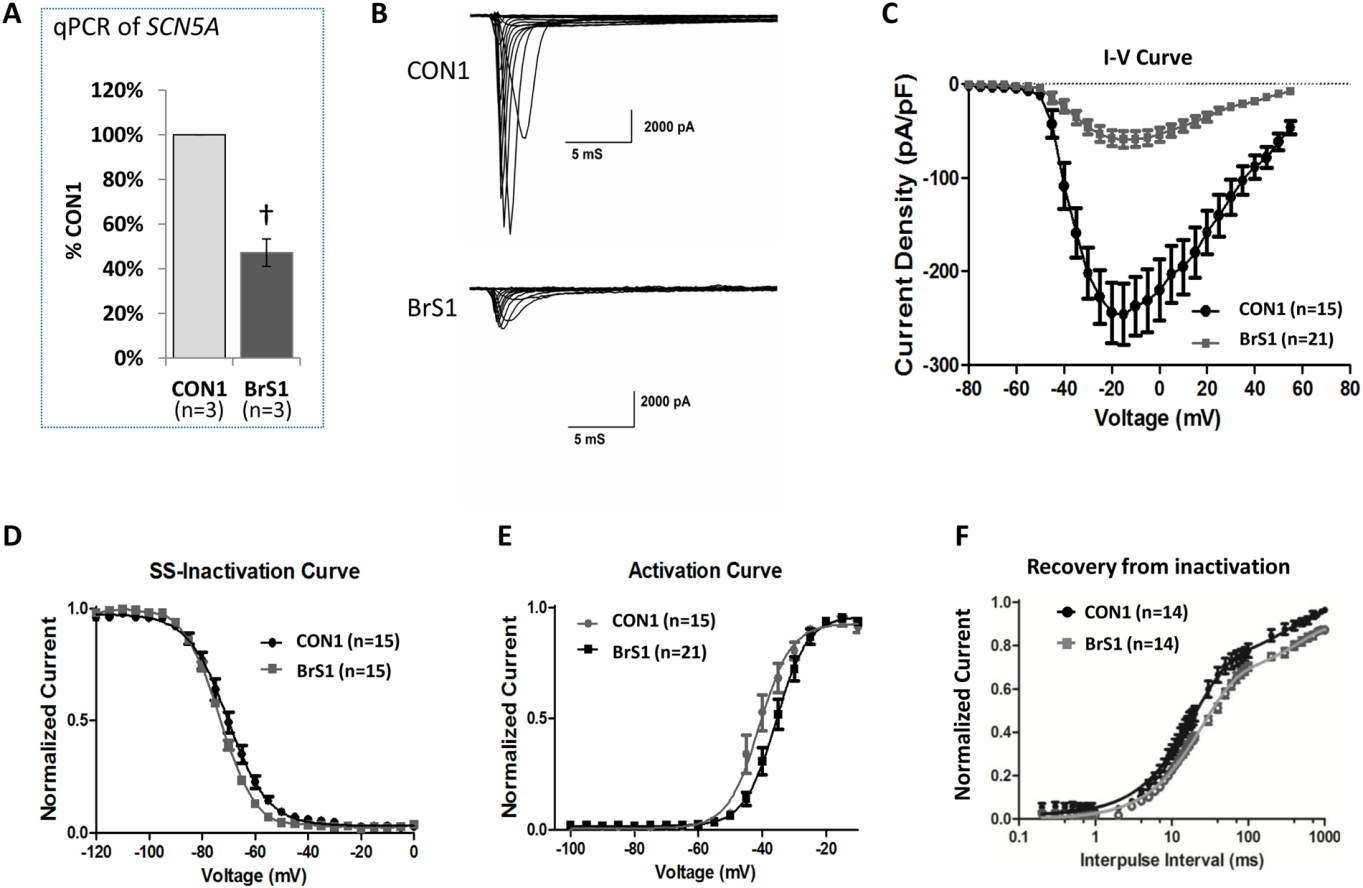
SCN5A and Nav1.5 expressions and *I*_Na_ measured in hiPSC-CMs. (**A**) Level of *SCN5A* mRNA in BrS1 (hiPSC-CMs from a BrS patient with compound *SCN5A* mutations) and CON1 (the sib of BrS1) determined by qPCR. n = 3. (**B**) Representative traces of sodium currents in the control (CON1) and patient (BrS1). (**C**) Peak *I*_Na_ density measured in BrS1 and CON1. (**D**–**F**) Steady state (SS)-inactivation, activation and recovery from inactivation curves of *I*_Na._ Values given are mean ± SEM. ^†^*p* < 0.01; vs. CON1.

Next, a ~50% reduction of the *SCN5A* mRNA was noted in hiPSC-CMs generated from the BrS patient (BrS1) compared with the sibling control (CON1) indicating a possible nonsense-mediated decay (Fig. 3A). The *I*_Na_ density measured in BrS1 was ~25% of CON1 (Fig. 3B,C). Relative to the CON1, BrS1 showed marginal/moderate changes in the rate of SS-inactivation and activation, and a more significant change in the rate of recovery from inactivation (Table S1, Fig. 3D–F).

### Identification of a loss-of-*I*_Na_ basal AP pattern with BrS1

HiPSC-CMs are known for their lack of *I*_K1,_ which is responsible for their depolarized resting membrane potential (RMP) of ~−60 mV compared with the ~−90 mV of quiescent adult ventricular and atrial myocytes^15,16^. The more positive resting membrane potential in hiPSC-CMs importantly results in inactivation of the fast *I*_Na_, leaving very limited *I*_Na_ available during the phase-0 depolarization and thereby potentially masking the impact of *I*_Na_ deficiency on the AP. The depolarized state of hiPSC-CMs could also lead to *I*_to_ inactivation and the absence of *phase-1 repolarization* in hiPSC-CMs^16^. Indeed, APs recorded from spontaneously contracting CON1 and BrS1, or BrS1 paced at 1.5, 1.0, 0.5, 0.2 Hz, were comparable without displaying a *phase-1 repolarization* morphology (Table S2, Figure S3).

**Figure 4:**
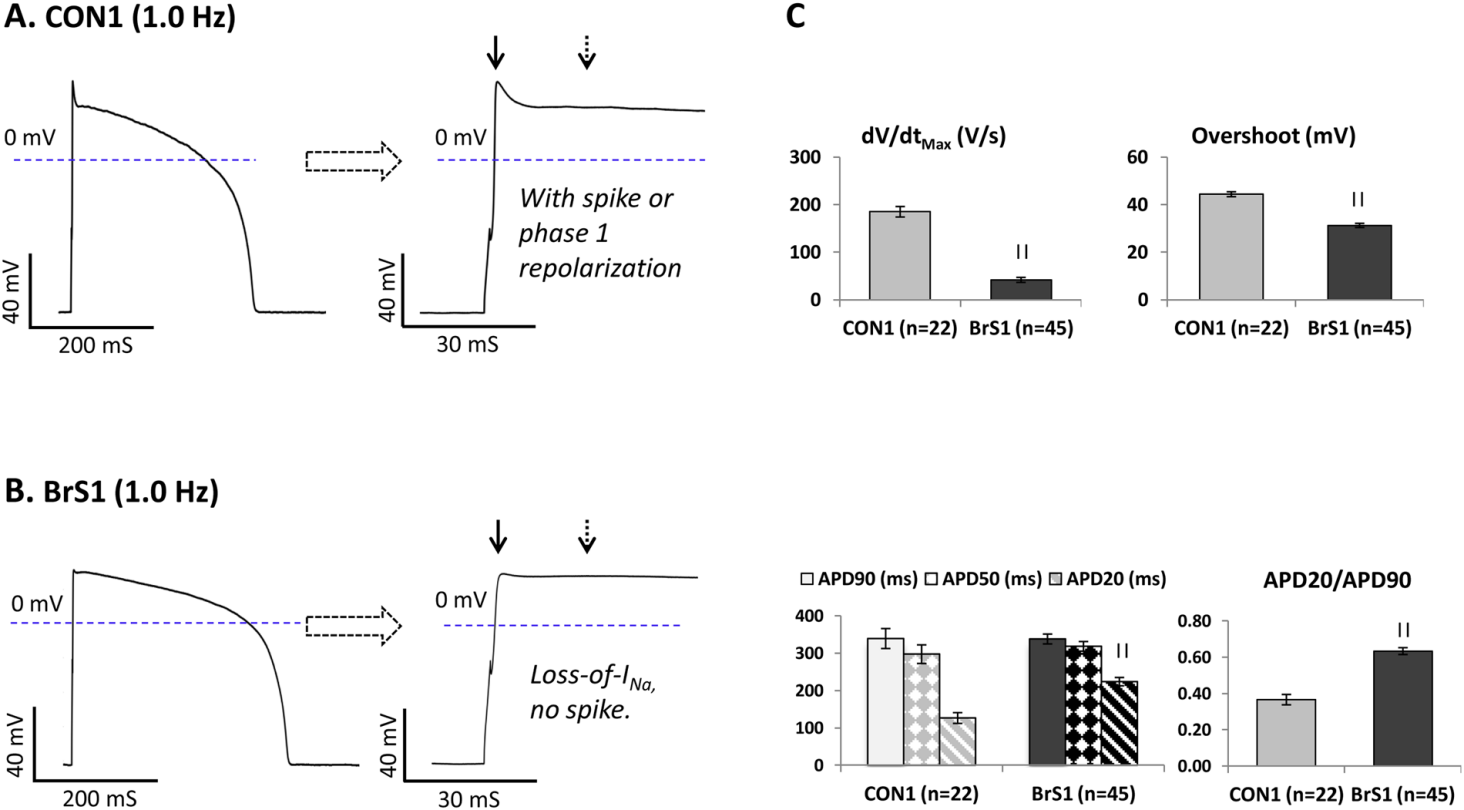
A basal action potential pattern of BrS identified in BrS1. (**A** and **B**) Representative AP waveforms recorded from a CON1 and a BrS1 paced at 1.0 Hz. The solid and dashed arrows indicate APA measured at phase-0 and phase-2, respectively. (**C**) The main AP parameters of CON1 and BrS1 are plotted and compared in bar-graphs. Values given are mean ± SEM. ^||^*p* < 0.00001; vs. CON1 (by unpaired *t*-test).

To overcome this limitation, a synthetic, cardiomyocyte membrane potential-dependent *in silico I*_K1_ was injected into hiPSC-CMs using the dynamic clamp technique^15^ to produce a physiologically polarized resting membrane potential (−84 mV). Thereafter, ventricular-like hiPSC-CMs (*I*_K1_^positive^) from CON1 (Fig. 4A) paced at a 1.0 Hz displayed a *phase-1 repolarization* AP morphology that resembles normal human ventricular myocytes. In contrast, an AP rounded pattern, clearly distinguishable from the *phase-1 repolarization* pattern of CON1, was observed in ventricular-like BrS1 (Fig. 4B). Such APs were characterized by over 75% reduction in the maximum upstroke velocity (dV/dt_Max_) and reduced AP amplitude (APA) and overshoot (Table S3, Fig. 4C). This AP pattern, referred as a “*loss-of-I*_Na_” AP morphology, reflects a marked *I*_Na_ deficiency in BrS1 during depolarization.

### Identification of a heart rate-induced *increased phase-1 repolarization* proarrhythmic AP change in BrS1

**Figure 5:**
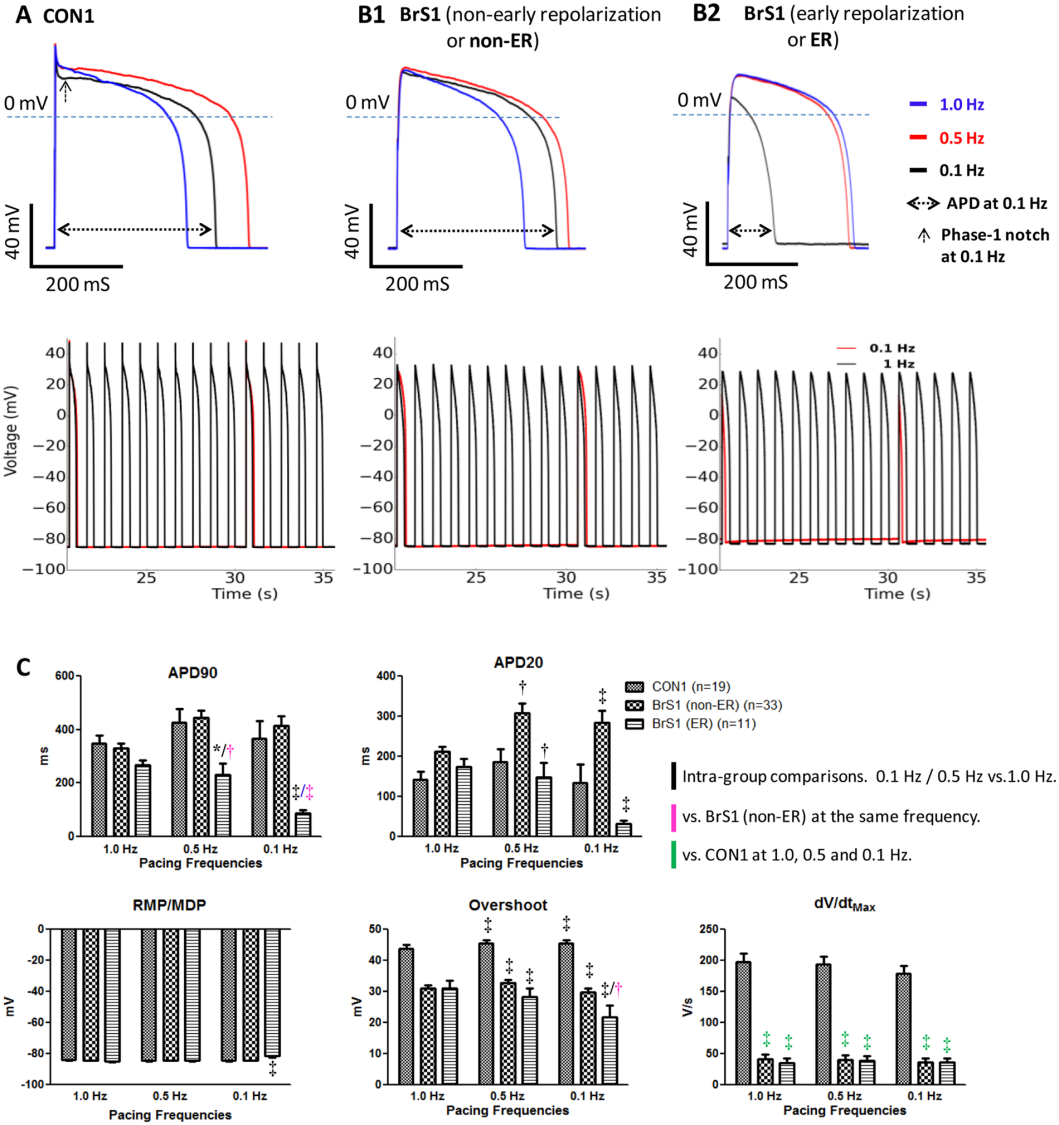
Effects of low heart rates on APs of BrS1 and CON1 hiPSC-CMs. (**A**, **B1** and **B2**) (Top): representative superimposed AP waveforms recorded from CON1 and two BrS1 subgroups (non-early repolarization and early repolarization), sequentially paced at 1.0 Hz, 0.5 Hz and 0.1 Hz. (**A**, **B1** and **B2**) (Bottom): superimposed AP waveforms continually recorded from CON1 and two BrS1 subgroups sequentially paced at 1.0 Hz and 0.1 Hz. (**C**) Main AP parameters plotted and compared among groups in bar-graphs. Values given are mean ± SEM. Statistics were performed with two-way repeated measures ANOVA followed by the Bonferroni post hoc testing. *p < 0.05; ^†^*p* < 0.01; ^‡^*P* < 0.001. Black symbols: Intra-(sub) group comparisons. 0.1 Hz/0.5 Hz vs. 1.0 Hz. Pink symbols: vs. BrS1 (non-ER subgroup) at the same frequency. Green symbols: vs. CON1 subgroup at 1.0, 0.5 and 0.1 Hz.

In BrS patients, sleep and rest are the most common triggers of ST-segment elevation, ventricular arrhythmia, and sudden death^1,3^. To test the effect of heart rate on the pathophysiology of BrS, APs were recorded from CON1 and BrS1 paced at 1.0 Hz, 0.5 Hz and 0.1 Hz. Responding to reduced pacing frequencies from 1.0 Hz to 0.1 Hz, CON1 showed slight changes in APD (±27% change in APD90 and the maximal shortening per cell was <33%) (Table S4, Fig. 5A, Table S5). In contrast, diverse changes in APD were observed with BrS1 paced at 0.1 Hz. The majority (75%) of BrS1 (the non-early repolarization subgroup) demonstrated a marked prolongation of APD90 or moderate shortening (<28% per cell) like CON1 (Fig. 5B1). However, a fraction (25%) of BrS1 (the early repolarization subgroup) displayed a drastic shortening of APDs (APD90 reduced by 66% in average and ≥50% per cell) accompanied by decreased dV/dt_Max_ and APA, and a more depolarized resting membrane potential (Table S4, Fig. 5B2, Table S5**)**. The marked reduction in APD, particularly the phase-2 APD indicated by an over 50% reduction in the ratio of APD20/APD90, represented an *increased phase-1 repolarization* AP pattern highly resembles the proarrhythmic *loss-of-dome* AP change recorded from canine right ventricle epicardium model under the *repolarization disorder hypothesis*^7,8^.

**Figure 6:**
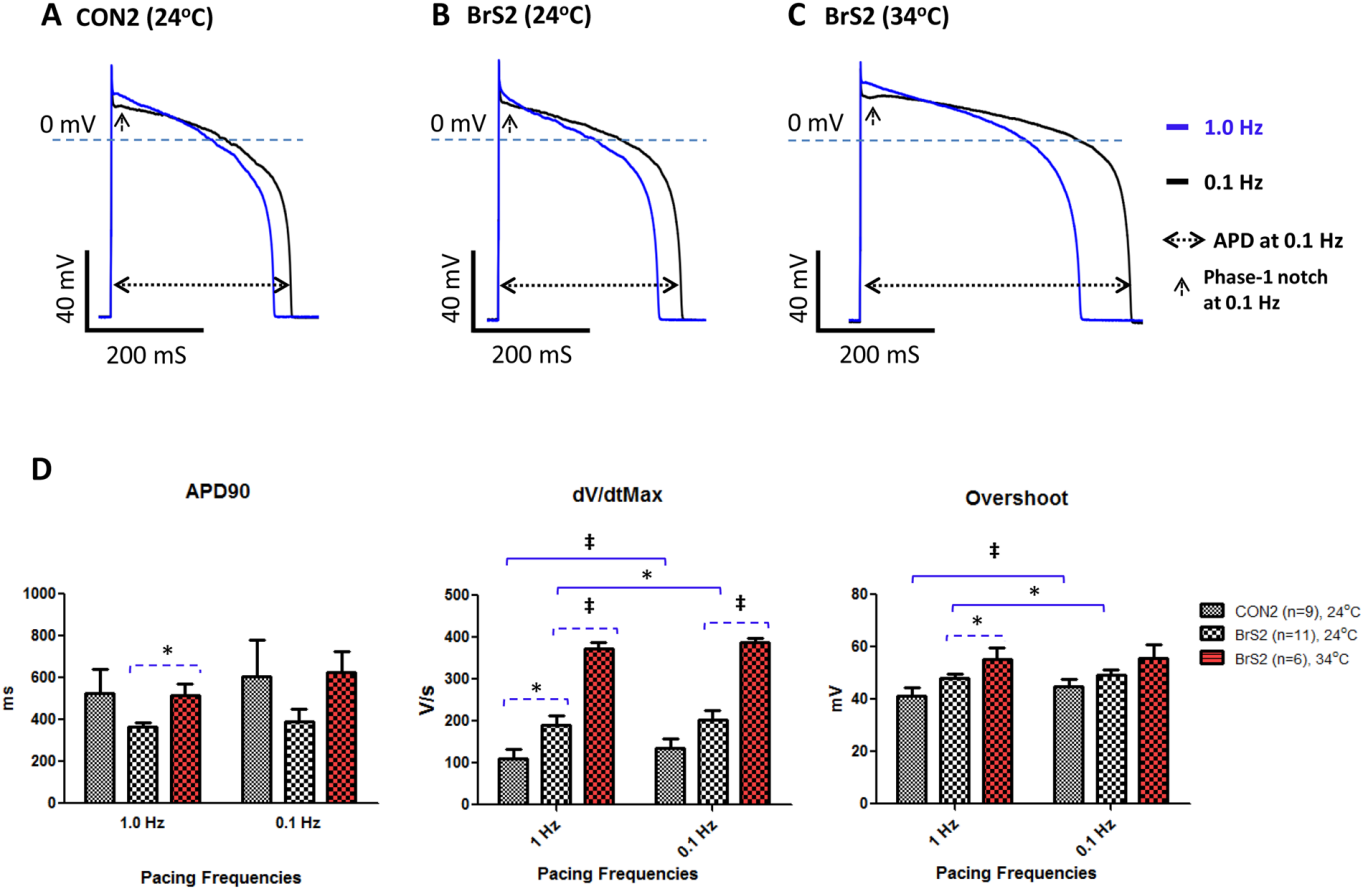
Validation of the rate-dependent effects on APs in BrS2 and CON2. (**A**) Representative superimposed AP waveforms of a CON2 cell paced at 1.0 and 0.1 Hz at 24 °C. (**B** and **C**) Representative superimposed AP waveforms of BrS2 cells paced at 1 and 0.1 Hz at 24 °C and 34 °C, respectively. (**D**) Main AP parameters are plotted and compared among groups in bar-graphs. Values given are mean ± SEM. Statistics were performed with two-way repeated measures ANOVA followed by the Bonferroni post hoc testing (by paired *t*-test). *p < 0.05; ^‡^*P* < 0.001.

On the other hand, CON2 and BrS2 (at both 24 °C and 34 °C) paced at 1.0 and 0.1 Hz displayed APDs within the normal range, similar to CON1. In agreement with that observed in tsa201 cells^17^, we observed an increased dV/dt_Max_ and APA/overshoot in hiPSC-CMs at 34 °C compared with that 24 °C, indicating an increased *I*_Na_ at 34 °C (Fig. 6). Normal levels of *I*_Na_ were observed with BrS2 and dV/dt_Max_ at 34 °C and 24 °C were all ≥190, which is greater than the 110~185 level of controls (CON1 and CON2) and significantly larger than the 41.9 ± 5.3 levels observed in BrS1.

### Evaluating the role of *I*_to_ in mediating the heart rate-dependent AP changes

In cardiac myocytes, Ca^2+^-independent *I*_to_ consists of a fast recovery component (*I*_to_,_f_) mediated mainly by Kv4.3 subunits and a slow recovery component (*I*_to_,_s_), mediated by Kv1.4 subunits. *I*_to_ contributes to the phase-1 repolarization of the AP. Slow heart rates favor the recovery of *I*_to_^18,19^, whereas increased *I*_to_ levels could accentuate the phase-1 notch of the AP^20–22^.

**Figure 7:**
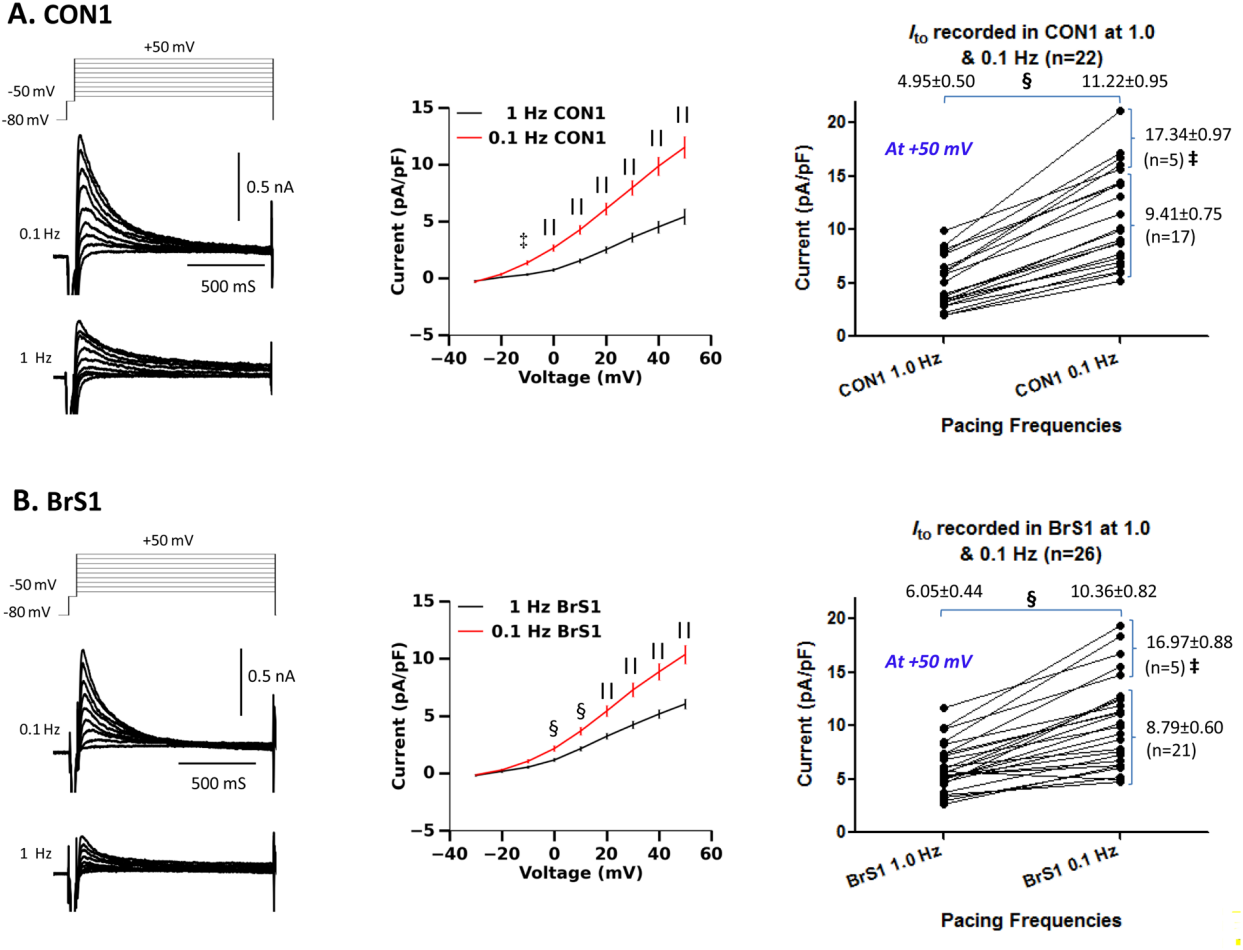
Effects of heart rates on *I*_to_ in hiPSC-CMs. (**A**-**left** and **B-left**) Representative traces of *I*_to_ currents recorded from CON1 (n = 26) and BrS (n = 22) paced at 1.0 Hz and 0.1 Hz. (**A-middle** and **B-middle)** I-V relationships of *I*_to_ show the current densities. (**A-right** and **B-right**) Graphic demonstrations and comparisons of *I*_to_ (recorded at +50 mV) in each cell are presented. Values given are mean ± SEM. ^§^*p* < 0.0001; ^||^*p* < 0.00001; vs. 1.0 Hz (by paired *t*-test and one-way ANOVA test). ^‡^*P* < 0.001, vs. low *I*_to_ group.

To explore the potential involvement of *I*_to_ in heart rate-induced AP changes, the level of *I*_to_ in CON1 and BrS1 paced at 1.0 Hz and 0.1 Hz were measured and compared (Fig. 7). *I*_to_ densities of both CON1 and BrS1 paced at 1.0 Hz were comparable (~5 pA/pF at 40 mV), which is **~**50% of that in isolated human sub-epicardial ventricular myocytes (10.6 ± 1.08 pA/pF at 40 mV)^18^. A ~100% increase (p < 0.00001) of *I*_to_ densities was recorded in both CON1 and BrS1 when the heart rate was reduced to 0.1 Hz. Moreover, variations in the levels of *I*_to_ among different hiPSC-CMs, particularly of those paced at 0.1 Hz, were identified in a fraction of cells (19~23%) which showed much higher *I*_to_ densities. Our data suggest potential heterogenic expression of *I*_to_ among hiPSC-CMs and *I*_to,s_ could be the dominant component of *I*_to_.

**Figure 8:**
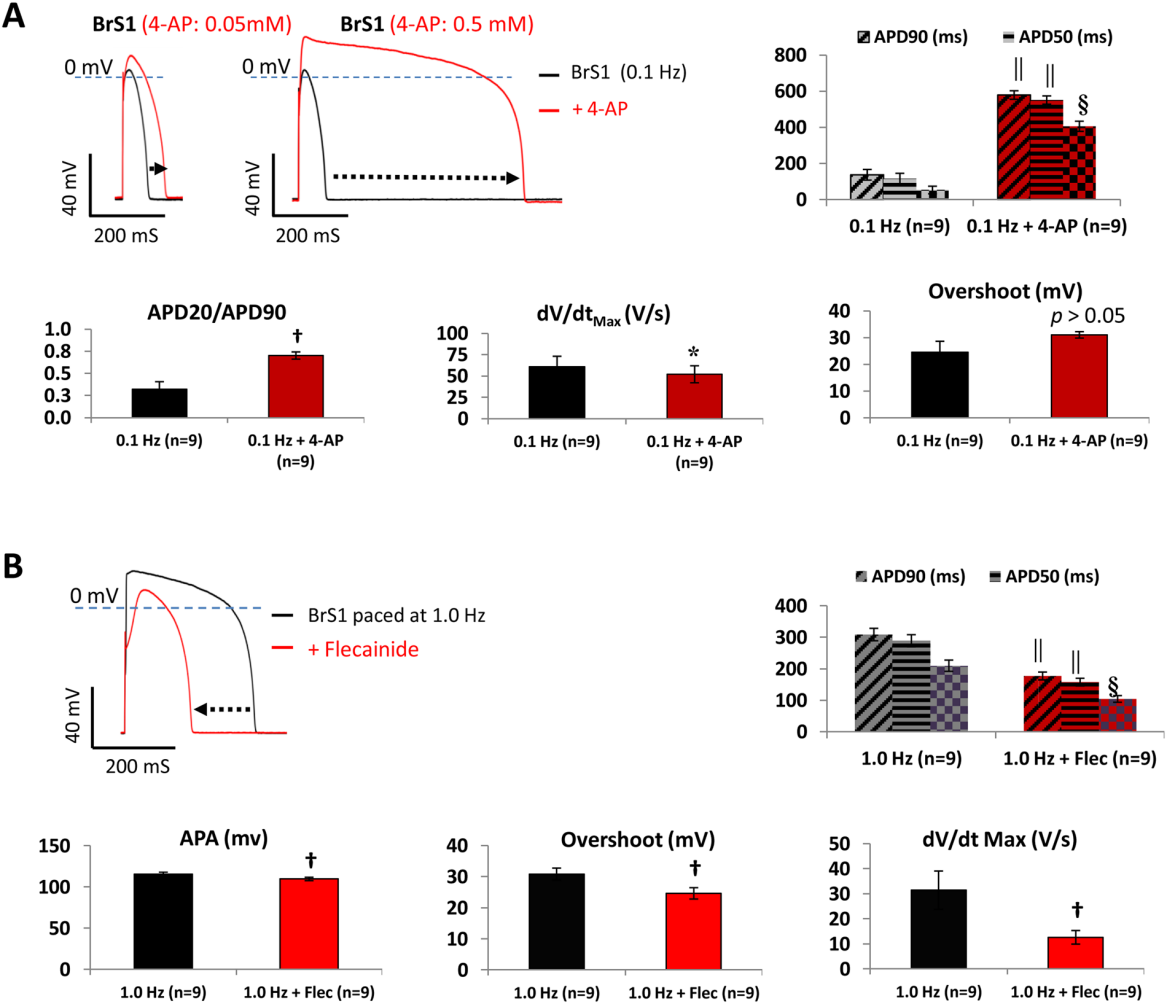
Effects of 4-AP and flecainide on APs. (**A**) Representative superimposed AP waveforms of a BrS1 cell paced at 0.1 Hz and subsequently exposed to 0.05 mM and 0.5 mM 4-AP. Main AP parameters (showing only the results of 0.5 mM 4-AP) are presented in bar-graphs. (**B**) Representative superimposed AP waveforms of a V-like BrS1 hiPSC-CM paced at 1.0 Hz followed by flecainide (10 µM) treatment. Main AP parameters are presented in bar-graphs. Values given are mean ± SEM. *p < 0.05; ^†^*p* < 0.01; ^§^*p* < 0.0001; ^||^*p* < 0.00001 (by paired *t*-test); vs. 0.1 Hz (for 4-AP) or 0.1 Hz (for flecainide).

To further validate the role of *I*_to_ in heart rate-induced AP changes, BrS1 that displayed an *increased phase-1 repolarization* AP change at 0.1 Hz were treated with 4-Aminopyridine (4-AP), which is known to block both *I*_to_,_f_ and *I*_to_,_s_^23,24^ at higher concentrations (0.5 mM to 10 mM could effectively block 55−85% of *I*_to_)^22^. As 4-AP at 1.0 mM and above could also block the rapid and slow components of the delayed rectifier K^+^ currents (*I*_Kr_ and *I*_Ks_) by over 25% and 39%, respectively^25^, we applied 0.05 mM and 0.5 mM 4-AP to BrS1 and noted that 0.05 mM 4-AP caused moderate APD prolongations, while 0.5 mM 4-AP completely reversed the *increased phase-1 repolarization* (Table S6, Fig. 8A). The significant increase in the ratio of APD20/APD90 suggested that 4-AP treatment recovered the loss of phase-2 repolarization.

### Blocking *I*_Na_ induced *increased phase-1 repolarization-like* change in BrS1

Ajmaline and flecainide are commonly employed in the clinical setting as challenges to attempt to unmask the characteristic BrS ECG phenotype and its associated arrhythmic events^7^. We observed that BrS1 paced at 1.0 Hz responded to flecainide (10 µM) with a significant shortening in APDs (with an average of 43.6% reduction in APD90) accompanied by reductions in dV/dt_Max_ from 31.4 ± 7.7 to 12.6 ± 2.7 V/s (*p* < 0.01) and APA/overshoot, confirming an *I*_Na_ inhibition (Table S7 and Fig. 8B**)**. Control cardiomyocytes (CON2) response to flecainide with reduced action potential amplitude/overshoot and dV/dt_Max_ similar to BrS1, indicating a spike and dome morphology change.

### Computer simulation of the effects of *I*_to_ and *I*_Ca,L_ on APDs

Adopting the O’Hara-Rudy model (2013) of human epicardial ventricular myocytes^26^, computer simulation was performed to examine the effects of *I*_to_ and *I*_Ca,L_ on APDs. While APD shortening was not seen in the presence of lower *I*_Na_ (*I*_Na_ reduced to 15%), it was observed with *I*_Na_ reduced to 15% (no effect with 20%) and *I*_to_ increased to 5 folds (Figure S5A). On the other hand, the shortened APD was not observed with reduced *I*_Ca,L_ in the presence of reduced *I*_Na_ (Figure S5B).

## Discussion

In cardiomyocytes derived from a BrS patient (BrS1) with a severe *SCN5A* haploinsufficiency, and an over 75% reduction in *I*_Na,_, we identified a highly distinguishable *loss-of-I*_*Na*_ AP pattern at normal pacing frequency (1.0 Hz) and an *increased phase-1 repolarization* AP pattern characterized by marked reduction in APDs and more depolarized resting membrane potential at a slow pacing frequency (0.1 Hz). Observation of these changes in repolarization required use of dynamic clamp to “electronically” express *I*_K1_ and allow recovery from inactivation of the major inactivating currrents *I*_Na_, *I*_Ca_ and *I*_to_. In contrast, cardiomyocytes without a marked *I*_Na_ reduction (BrS2) and two normal controls (CON1 and CON2) failed to show heart rate-induced AP abnormalities. Importantly, similar low levels *I*_Na_ were observed in four separate hiPSC lines from the same patient (Table S1), and one line was selected for our experiments. We further correlated the reduced heart rate (from 1.0 Hz to 0.1 Hz) with a ~100% increase in *I*_to_, suggesting that a higher *I*_to_ acts together with a low *I*_Na_ contributes to the APD shortening phenotype in BrS1.

Thus, the results from the current study suggest repolarization abnormalities. The repolarization hypothesis of BrS implies that the *loss-of-dome* change is limited to epicardial myocytes and corresponds to a transmural dispersion of repolarization causing an ST-segment elevation in the ECG^7^. The fact that cardiac myocytes from a BrS patient predisposed with a severe *I*_Na_ deficiency (such as BrS1) could develop arrhythmic changes at slow heart rates, and also associated with an elevated *I*_to_, supports the *repolarization disorder hypothesis* that a low *I*_Na_ in conjunction with a high *I*_to_ could result in an *increased phase-1 repolarization*^7,8^.

Interestingly, the heart rate-sensitive and *I*_to_-mediated basal and pro-arrhythmogenic AP phenotypes identified in BrS hiPSC-CMs might mirror a switch between the daytime/active/asymptomatic and sleep/bradycardia/disease onset states seen in many BrS patients. Even with a severe *I*_Na_ deficiency, APs of BrS1 remain largely ‘normal’ at normal heart rates which keep patients in ‘event-free’ clinical status. A precipitating factor is needed, such as sodium channel blockade^7^, to trigger or unmask arrhythmic events. *I*_to_, coordinated by *I*_Na_ and *I*_Ca,L_, plays a key role in shaping the AP of ventricular epicardial myocytes. *I*_to,f_ and *I*_to,s_^23,24^ coexist in ventricular myocytes and are distinguishable by the either tens or thousands of milliseconds taken for recovery from inactivation^23^. The expressions of Kv1.4 and Kv4.3 genes in human right ventricular myocytes have been confirmed, and the level of Kv1.4 is ~37.5% that of Kv4.3 and the Kv4.3-dependent KChIP2 is >7-fold that of Kv4.3^27^. *I*_to,s_ plays a role in ventricular myocytes and slow heart rates could facilitate its recovery from inactivation._._ An accentuated phase-1 notch in canine epicardial ventricular myocytes paced at 0.125 Hz ^19^and 0.5 Hz^20^, and in ferret ventricle myocytes paced at 0.2 Hz^23^, was noted. Moreover, canine ventricular myocytes paced at 0.125 Hz were more vulnerable to an ischemia-induced *loss-of-dome* AP change^28^. Furthermore, it has been shown that hiPSC-CMs responded to 1.0 Hz, 0.5 Hz and 0.1 Hz with 20%, 40% and 100% recovery of *I*_to,_ respectively; and hiPSC-CMs express a relatively high level of Kv1.4 (~80% of Kv4.3) and a much lower level of KChIP2 compared Kv4.3^28^. Our data from hiPSC-CMs are consistent with that of Cordeiro *et al*.^28^. and indicate that *I*_to,s_ could be a main component of *I*_to_ in hiPSC-CMs and play a dominant role at 0.1 Hz when there is more time to recover from inactivation. Our data further suggest that, like that among different subtypes of human ventricular myocytes such as those from the right ventricle pericardium and endocardium, *I*_to_ could be heterogeneously expressed among hiPSC-CMs and a small fraction of cells that express a distinguishable higher level of *I*_to_ could behave like the right ventricle epicardium myocytes prone to the ‘*loss-of-dome*’ AP change (Fig. 7). Treatment with 4-AP also reversed the increased phase-1 repolarization which we attribute to block of *I*_to_ channels (rather than potentiation of *I*_Ca,L_ which was reported in heterologous experiments^29^.

Our experimental findings from hiPSC-CMs are supported by results from a computer simulation study using the O’Hara-Rudy non-diseased human ventricular epicardial myocyte model (2011)^26^. When *I*_Na_ was reduced to 10~30% of the control level, a subsequent 1.7~2.4-fold *I*_to_ increments was associated with ‘*loss-of-dome*’ AP change^30^. Ca^2+^ handling was not investigated in our study, but has been reported to have a role in arrhythmogenesis in an hiPSC-CM model of Brugada^13^.

Although BrS patient-derived hiPSC-CMs represent the most advanced model that reflects the basal cellular phenotype associated with *SCN5A* defects and *I*_Na_ deficiency, further dissecting the mechanism behind the BrS is met with limitations. Beside a weaker than normal inward rectifier current (*I*_K1_) even when *I*_K1_ is increased using dynamic clamp, these cells have lower expression of slowly reactivating *I*_to_. Thus, hiPSC-CMs do not fully recapitulate the epicardial action potential phenotype, and the spike and dome configuration of the AP is not obvious.

Despite our novel and compelling findings, our study has limitations. First, our data may not fully explain the mechanisms behind many BrS cases in which the *SCN5A* defects are associated with only a milder/moderate *I*_Na_ deficiency. Indeed, our data from BrS2 demonstrated that the heterologous T1620M variant^5,17^ shows a minimal, if any, impact on *I*_Na_ in hiPSC-CMs recorded at both 24 °C and 34 °C, reflected by the normal dV/dt_Max_ and APA/overshoot. A recent report further supports this notion by showing normal APs from the hiPSC-CMs of BrS patients without any genetic mutations^12^. The likely different expression profiles of *I*_to,f_ and *I*_to,s_ in hiPSC-CMs (*I*_to,s_ –dominant, demonstrated in this study and a previous report^28^) compared with that in human epicardial ventricular myocytes (*I*_to,f_ –dominant^23,24^) may compromise the value of this model. This may also explain the lack of consistency between the cellular phenotypes and the clinical findings of the patients. Second, we acknowledge that we did not correct these mutations with gene editing in the hiPSC-CMs to rescue the disease phenotype. However, we believe that using a healthy sibling and a standard cell line are appropriate controls in this study, as done previously by our lab and others^31–35^. Finally, although hiPSC-CMs were studied a longer than 30 days after differentiation, prolonged culture times can have effects on *I*_Na_ properties^36^. These effects should be taken into account when interpreting the data and further studies are needed to access the potential impact on hiPSC-CMs as an electrophysiological model.

## Conclusions

Using hiPSC-derived cardiomyocytes from a BrS patient with *SCN5A* mutations, we demonstrate that a severe *I*_Na_ deficiency could lead to a remodeled baseline APs vulnerable to heart rate-induced, *I*_to_-sensitive proarrhythmic *increased phase-1 repolarization* changes. Our data support a coordinated role of *I*_Na_ and *I*_to_ in the mechanism of BrS.

## Materials and Methods

For a full description of the materials and methods, see Supplemental Material.

### hiPSC-CMs from BrS patients and controls

To obtain a BrS hiPSC-CM model with marked *I*_Na_ deficiency, a BrS patient (II-2) with compound heterozygous mutation in *SCN5A* (a missense mutation: c.677 C > T, *p.*A226V, and a nonsense mutation: c.4885 C > T, *p*.R1629X)^14^, and a sibling control, were included in this study (Figure S1). All experimental protocols were approved by the institutional review board (Institutional Review Board of SingHealth, Singapore) and all methods were performed following the relevant guidelines and regulations. Clinical data and biological samples were taken following written informed consent. hiPSCs were generated from the dermal fibroblasts of the BrS patient and sibling control (Fig. 1). All hiPSC lines were expanded as adherent cultures in feeder-free conditions on Matrigel-coated dishes in the presence of chemically defined medium (E8 Essential Medium, Life Technologies). Differentiation of hiPSC to cardiomyocytes (CMs) was performed following a previously reported protocol based on small molecules-mediated canonical Wnt pathway modulation^37^. Four hiPSC clones, which showed good self-renewal potential and cardiac differentiation efficiency, were selected. Eventually, single CON1 and BrS1 clone, evidenced by yielding >55% cardiac troponin T (cTnT) positive hiPSC-CMs quantified by fluorescence-activated cell sorting (FACS) analysis (Figure S6), were adopted. HiPSC-CMs were further stained with anti-α-actinin, anti-β-MHC and anti-cardiac titin antibodies (Fig. 1D). The BrS1 and CON1 hiPSC-CMs were dissociated into single cells 35–45 days after cardiac differentiation and used for electrophysiology assays.

We also used cardiomyocytes (CON2), a commonly used control hiPSC line (iCell® Cardiomyocytes, from a female Caucasian with the age unknown), and a genome edited BrS hiPSC-CM line (BrS2) which carries the *p*. T1620M *SCN5A* mutation known to have a milder impact on *I*_Na_ in heterologous expression systems^4,6,17^. Both lines were commercially obtained from Cell Dynamic International (Madison, WI, USA).

### Whole-cell patch-clamp assays

An Axopatch 200B Amplifier controlled by pClamp10 software together with a Digidata 1440 acquisition system (Molecular Devices, Sunnyvale, USA) was used to record ionic currents and APs.

*I*_Na_ in tsa201 cells and hiPSC-CMs^32^, *I*_to_^12,24,28^ and the ultra-rapid delayed rectifier K^+^ current (*I*_Kur_)^38,39^ in hiPSC-CMs were measured using standard voltage-clamp protocols.

Dynamic clamp was performed using a Cybercyte System from Cytocybernetics (Buffalo, USA)^15^. With an injection of a synthetic *I*_K1_ current, the ventricular-like hiPSC-CMs (*I*_K1_^positive^) were defined as having AP amplitude (APA) over 100 mV, APD at 90% repolarization (APD90) over 200 ms and lack of *I*_Kur_^38^, which is highly expressed in the atrial-like human embryonic stem cell-derived cardiomyocytes^34^ and hiPSC-CMs (Figure S2).

### Statistical analyses

Numerical data are presented as mean ± standard deviation (SD) or mean ± standard error of the mean (SEM). Comparisons were made with unpaired and paired (two-tailed) Student’s *t*-test, one-way repeated measures ANOVA followed by the Tukey’s post hoc testing, and two-way repeated measures ANOVA followed by the Bonferroni post hoc testing using GraphPad Prism 5.0 (GraphPad Software, La Jolla, USA). A *p-*value of <0.05 was considered statistically significant.

## Electronic supplementary material

Supplementary Information
