# Supplementary material for: Identification of an INa-dependent and Ito-mediated proarrhythmic mechanism in cardiomyocytes derived from pluripotent stem cells of a Brugada syndrome patient
Source: Sci Rep. 2018 Jul 26;8:11246. doi: 10.1038/s41598-018-29574-5 (PMC6062539; doi:10.1038/s41598-018-29574-5)
Supplement: Supplementary file 1 — Supplementary Information [file 41598_2018_29574_MOESM1_ESM.docx]

**Identification of an *I*_Na_-dependent and *I*_to_-mediated proarrhythmic mechanism in cardiomyocytes derived from pluripotent stem cells of a Brugada syndrome patient**

Dongrui Ma, Zhenfeng Liu, Li Jun Loh, Yongxing Zhao, Guang Li, Reginald Liew, Omedul Islam, Jianjun Wu, Ying Ying Chung, Wee Siong Teo, Chi Keong Ching, Boon Yew Tan, Daniel Chong, Kah Leng Ho, Paul Lim, Rita Yu Yin Yong, Brian K. Panama, Aaron D. Kaplan, Glenna CL Bett, James Ware, Connie R. Bezzina, Arie O. Verkerk, Stuart A. Cook, Randall L. Rasmusson and Heming Wei

**Supplemental methods**

1. **The BrS patient**

A Chinese family with BrS was identified (Figure S1A**)**.**^1^** The patient (II-2) presented with recurrent syncopal events at the age of 11 with spontaneous Type 1 Brugada (coved type) ECG recorded at the V2 lead (Figure S1B).**^1^** Compound heterozygous mutations in the SCN5A gene (NM_198056.2), including a missense c.677C>T (p.Ala226Val or A226V) and a nonsense c.4885C>T (p. Arg1629X or R1629X) mutation, were detected in the proband (II-2). Both mutations are located in the highly conserved regions at the 4^th^ segments (positive charged, essential as voltage sensors) of the 1^st^ (I) and 4^th^ (IV) transmembrane domains of Na_v_1.5 (Figure S1C). The father (I-1) and the sister (II-3) carry the A226V mutation without symptoms. The mother (I-2) carries the R1629X mutation and her ECG shows BRRR with a broader QRS yet free of symptoms. Genomic DNA was extracted from the peripheral blood and genotyping was performed at Clinical Genetics, AMC.

1. **Generation of hiPSCs via reprogramming of dermal fibroblasts**

A 5-mm skin punch biopsy was taken from the proband (II-2) and his mutation-free, symptom-free older brother (II-1) who served as sibling control. Dermal fibroblasts were derived and reprogrammed by repeated transfection of a set of synthetic modified mRNA of OCT-4, SOX-2, KIF-4, c-Myc, and LIN28 (Stemgent, Cambridge, USA).**^2^**

Standard characterizations of hiPSCs were conducted as previously described.**^2^** The pluripotency of hiPSCs was characterized *in vitro* by immunofluorescent assay for their expression of human pluripotent stem cell markers (Figure S2A) and in vivo by teratoma formation assay (Figure S2B). The genetic stability of hiPSCs was validated by Karyotyping (Figure S2C).

1. **Generation of hiPSC-CMs via cardiac differentiation**

Well characterized control and BrS hiPSC lines were differentiated into cardiomyocytes.**^3^** In brief, monolayers of single hiPSC single cells dissociated from hiPSC colonies were prepare. The differentiation of hiPSCs was carried out in a serum-free defined system by temporal modulation of canonical Wnt signaling: appropriate temporal application of Gsk3 inhibitor (CHIR99021) followed by chemical Wnt inhibitors (IWP).

Contracting clusters of hiPSC-CMs were dissociated by incubating with TrypLE™ reagents (Life Technologies, Singapore) at 37^o^C in a CO_2_ incubator for 5-8 minutes to become single cells. To validate the cardiac differentiation efficiency, flow cytometry was performed to determine the percentage of cardiac Troponin T (cTnT) positive hiPSC-CMs in our cardiac differentiation culture. About 2×105 cells were stained with mouse anti-human cTnT antibody (Millipore) while cells stained with mouse IgG1 isotype control antibody (Millipore) were used as negative control. Experiments were repeated twice with duplicates for each sample. Next, hiPSC-CMs were replated on 0.1% gelatin-coated cultural plastic or glass surface. The identity and structural integrity of hiPSC-CMs from patient and control were determined by immunofluorescence staining of cardiac sarcomeric proteins including cardiac sarcomeric α-actinin, myosin heavy chain β (β-MHC) and titin for their expression and intracellular patterns. hiPSC-CMs were fixed in 4% paraformaldehyde, permeabilized in 0.1% Triton X-100 and incubated with Monoclonal antibodies against cardiac sarcomeric α-actinin (clone EA-35. Sigma), β-MHC (Alexis Biochemicals, FL, USA), and cardiac titin (1:10) (Sigma-Alrich, MO, USA) at 4^o^C overnight. Next, cells were incubated with Alexa Fluo® 488 goat anti-rabbit IgG or Alexa Fluo® 555 donkey anti-rabbit IgG (Invitrogen, CA, USA). Fluorescent images were viewed by a [Laser Scanning Confocal Microscope](http://microscopy.zeiss.com/microscopy/en_de/products/confocal-microscopes.html) (Carl Zeiss LSM 710, Germany).

1. **Heterologous expression of SCN5A in tsa201 cells**

A cDNA clone of human SCN5A (NM_198056.2) was obtained from OriGene (Rockville, MD, USA). Using it as template, the c.677C>T (A226V) and the c.4885C (R1629X) mutations were separately created using QuikChange XL Site-Directed Mutagenesis Kit (Agilent Technologies, Santa Clara, USA). Next, equal amount of the wild type (WT)-, A226V- and the R1629X-SCN5A expressing vectors were co-transfected with hβ1-subunit cDNA into tsa201 cells using Lipofectamine® 2000 Transfection Reagent (Life Technologies, Singapore).

1. **Expression of SCN5A and Na_v_1.5 determined by qPCR, immunofluorescence and Western blotting assays**

The expression of SCN5A mRNA in hiPSC-CMs was measured by quantitative RT-PCR (qPCR). First strain cDNA was synthesized from total RNA isolated from control and BrS hiPSC-CMs and amplified in Platinum SYBR Green qPCR Supermix (Roche Applied Science, Switzerland) using the Lightcycler TM 480 real-time PCR system (Roche, Switzerland). The PCR primers for SCN5A were: Forward: 5’ ACATCATCATCTCCTTCCTCAT -3’ and Reverse: 5’-CATATCGAAGTCGTCCTCAC- 3’. The PCR amplified a product of 116 base pairs downstream of the c.4885C>T nonsense mutation. PCR products of SCN5A were adjusted with that of beta-actin.

The levels and intracellular localizations of Na_v_1.5 in transfected tsa201cells were determined by immunofluorescent staining. Cells were fixed in 4% paraformaldehyde, permeabilized in 0.1% Triton X-100 and incubated with a rabbit-anti human anti-Na_v_1.5 antibody (#ASC-005, Alomone labs, Jerusalem, Israel). Fluorescent images were viewed with laser scanning confocal microscopy (Carl Zeiss LSM 710, Germany). Quantification of Na_v_1.5 was performed with Image J software (NIH).

The sizes of Na_v_1.5 protein in transfected tsa201 cells (with transfection efficiency over 70%) and hiPSC-CMs were determined by Western blotting with a rabbit-anti human anti-Na_v_1.5 antibody (#ASC-005, Alomone labs, Jerusalem, Israel).

1. **Electrophysiological recordings**

Ion currents and action potentials (APs) were measured in dissociated single hiPSC-CMs using a Axopatch 200B patch clamp amplifier (Axon patch 200B, Axon Instruments, Sunnyvale, USA). Data acquisition was achieved using the Digidata 1440 (Axon instrument) and signals were low-pass filtered at 5 kHz. Recordings were analyzed using the pClamp10.7 software (Clampex, Axon Instruments) and Origin 7.0 software (Origin Lab Corporation). To determine the ion current density, ion currents were normalized to the cell capacitance (Cm(pF) which is automatically measured using the pClamp10.7 software after compensations. To record the fast sodium currents (*I*_Na_), the transient outward potassium (*I*_to_) and the ultra-rapid delayed rectifier potassium (*I*_Kur_) currents, the series resistance and cell capacitance were compensated to 75%.

**6.1. Action potential recordings in hiPSC-CMs**

Both spontaneous and paced (at 1.0 Hz) APs were recorded at 34^o^C following conventional current-clamp protocol.**^2^**  The pipette solution contained (in mM): KCl 140, NaCl 10, MgCl_2_ 1, MgATP 5, Na_2_GTP 0.2, EGTA 5 and HEPES 10, adjusted to pH 7.2 with KOH. Extracellular solution contained (in mM): NaCl 140, KCl 5.4, CaCl_2_ 1.8, MgCl_2_ 1, glucose 10 and HEPES 10, adjusted to pH 7.4 with NaOH. AP signals were low-pass filtered at 5 kHz and digitized at a sampling rate of 20 kHz.

The dynamic action potential clamp (DAPC) technique was used to inject an *in silico* inward rectifier K^+^ current (*I*_K1_) using the Cybercyte System (Cytocybernetics, Buffalo, USA) as previously described.**^4^** APs were recorded with DAPC technique at 24^o^C and in some cases at 34^o^C. The synthetic *I*_K1_ is generated in real time in response to the cardiomyocyte membrane potential. For hiPSC-CMs used in this study, the potentiometer was set to provide a standard outward current peaked at 150 pA at −75 mV.

**6.2. *I*_Na_ recordings**

*I*_Na_ was recorded in tsa201 cells and hiPSC-CMs at room temperature (24^o^C) using the voltage clamp technique.**^2, 5^** Currents were digitized at a sampling rate of 5 kHz. Patch pipettes (borosilicate glass, 2–3 MΩ) were filled with solution contained (in mM): CsCl 133, NaCl 3, MgCl_2_ 2, EGTA 10, HEPES 5, TEA-Cl 2 and Na_2_-ATP 4, adjusted to pH 7.2 with CsOH. The external solution contained (in mM): NaCl 135, CsCl 4.2, MgCl_2_ 1.2, CaCl_2_ 1.8, HEPES 10 and glucose, adjusted to pH 7.4 with CsOH.

For recording activation and inactivation of *I*_Na_, currents were measured at a cycle length of 500 ms from a holding potential of -90 mV. Voltage dependence of activation was estimated by measuring peak Na^+^ current during a variable test potential from a holding potential of -90 mV with 30 ms pulses of increasing amplitude (5 mV steps) from -100 mV to +10 mV. Steady state inactivation was measured by a double pulse protocol from a holding potential of -90 mV consisting of a 50 ms pulse of increasing amplitude from -120 mV to +10 mV (5 mV steps) followed by a test pulse of 30ms to -20 mV. Steady-state activation and inactivation curves were fit using the Boltzmann equation I/Imax=A/{1.0+exp[(V_1/2_-V)/κ]} to determine V_1/2_ (membrane potential for the half-maximal (in)activation) and the slope factor κ.

Recovery from inactivation of *I*_Na_ was measured by a double pulse protocol using depolarization pulses from -90 mV to-20 mV and interpulse intervals ranging from 0.1 to 1000 ms. Recovery from inactivation was analyzed by fitting a double-exponential function to the data to obtain the time constants of the fast and the slow components of recovery from inactivation I/Imax = A_f_ * [1.0-exp(-t/τ_f_)] + A_s_* [1.0-exp(-t/τ_s_)], where t is the recovery time interval, τ_f_ and τ_s_ are the time constants of the fast and slow components, and Af and As are the fractions of the fast and slow components, respectively.

**6.3. *I*_Kur_ currents measurements**

*I*_Kur_ currents were measured in CON2, a normal hiPSC-CMs line (iCell® Cardiomyocytes) obtained from Cellular Dynamics International (Madison, WI, USA)**^5^** at 24^o^C by depolarizing voltage steps from a holding potential of –80 mV to +50 mV.**^6,7^** A prepulse from an holding potential positive to -20 mV was to inactivate *I*_to_ current.**^7^** The calculated liquid junction potential is small and was not corrected. The same pipette solution as for AP recording was adopted whereas the extracellular solution was modified from that for AP recording by reducing extracellular [Ca^2+^]_i_ to 0.5 mM and addition of 100 µM CdCl_2_ so to eliminate the Ca^2+^ current.**^6^**

To validate the identity of a ventricular-like hiPSC-CM, an estimated *I*_Kur_ current, presented as an outward current at the end of AP repolarization phase, was used as an index in addition to APD90. It was noticed that this current was strong in cells with atrial-like AP morphology and shorter APD90 (< 200 ms), whereas it was much smaller in cells with ventricular-like AP morphology and longer APD90 (≥ 200 ms). See Figure S3.

**6.4. *I*_to_ currents measurements**

*I*_to_ currents were measured in CON1 and BrS1 at 24^o^C by depolarizing voltage steps from a holding potential of –80 mV.**^8^** The cycle lengths were 1000 ms (1 Hz) and 10,000 ms (0.1 Hz). A prepulse of 10 ms to -40 mV was used to inactivate *I*_Na_. The calculated liquid junction potential is small and was not corrected. The same pipette solution as for AP recording was adopted whereas the extracellular solution was modified from that for AP recording by addition of 500 µM CdCl_2_ so to eliminate the Ca^2+^ current.**^1^**

**7. Drugs**

Both 4-Aminopyridine (4-AP) and flecainide acetate salt were obtained from Sigma-Aldrich (St. Louis, USA). The stock solution of 4-AP (5 mM) and flecainide (10 mM) were freshly prepared in Tyrode’s solution.

**References:**

1. Tan BY, Yong RY, Barajas-Martinez H, Dumaine R, Chew YX, Wasan PS, Ching CK, Ho KL, Gan LS, Morin N, Chong AP, Yap SH, Neo JL, Yap EP, Moochhala S, Chong DT, Chow W, Seow SC, Hu D, Uttamchandani M, Teo WS. A brugada syndrome proband with compound heterozygote scn5a mutations identified from a chinese family in singapore. *Europace*. 2016; 18:897-904. doi:10.1093/europace/euv058

2. Ma D, Wei H, Zhao Y, Lu J, Li G, Sahib NB, Tan TH, Wong KY, Shim W, Wong P, Cook SA, Liew R. Modeling type 3 long qt syndrome with cardiomyocytes derived from patient-specific induced pluripotent stem cells. *Int J Cardiol*. 2013;168:5277-5286. doi: 10.1016/j.ijcard.2013.08.015

3. Lian X, Hsiao C, Wilson G, Zhu K, Hazeltine LB, Azarin SM, Raval KK, Zhang J, Kamp TJ, Palecek SP. Robust cardiomyocyte differentiation from human pluripotent stem cells via temporal modulation of canonical Wnt signaling. *Proc Natl Acad Sci U S A.* 2012;109: E1848-1857. doi:10.1073/pnas.1200250109

4. Bett GC, Kaplan AD, Lis A, Cimato TR, Tzanakakis ES, Zhou Q, Morales MJ, Rasmusson RL. Electronic "expression" of the inward rectifier in cardiocytes derived from human-induced pluripotent stem cells. *Heart rhythm*. 2013;10:1903-1910. doi:10.1016/j.hrthm.2013.09.061

5. Ma J, Guo L, Fiene SJ, Anson BD, Thomson JA, Kamp TJ, Kolaja KL, Swanson BJ, January CT. High purity human-induced pluripotent stem cell-derived cardiomyocytes: electrophysiological properties of action potentials and ionic currents. *Am J Physiol Heart Circ Physiol*. 2011;301:H2006-2017. doi:10.1152/ajpheart.00694.2011

6. Amos GJ, Wettwer E, Metzger F, Li Q, Himmel HM, Ravens U. Differences between outward currents of human atrial and subepicardial ventricular myocytes. *J Physiol*. 1996;491:31-50. doi: 10.1113/jphysiol.1996.sp021194

7. Wang Z, Fermini B, Nattel S. Sustained depolarization-induced outward current in human atrial myocytes. Evidence for a novel delayed rectifier K^+^ current similar to Kv1.5 cloned channel currents. *Circ Res*. 1993;73:1061-1076. doi: 0.1161/01.RES.73.6.1061

8. Cordeiro JM, Nesterenko VV, Sicouri S, Goodrow RJ, Jr., Treat JA, Desai M, Wu Y, Doss MX, Antzelevitch C, Di Diego JM. Identification and characterization of a transient outward k+ current in human induced pluripotent stem cell-derived cardiomyocytes. *J Mol Cell Cardiol*. 2013;60:36-46. doi:10.1016/j.yjmcc.2013.03.014

9. Ivashchenko CY, Pipes GC, Lozinskaya IM, Lin Z, Xiaoping X, Needle S, Grygielko ET, Hu E, Toomey JR, Lepore JJ, Willette RN. Human induced pluripotent stem cell-derived cardiomyocytes exhibit temporal changes in phenotype. Am. J. Physiol. Heart Circ. Physiol. 2013;305: H913-H922. doi: 10.1152/ajpheart.00819.2012

10. Priori SG, Napolitano C, Di Pasquale E, Condorelli G. Induced pluripotent stem cell-derived cardiomyocytes in studies of inherited arrhythmias. J Clin Invest. 2013;123:84-91. doi: 10.1172/JCI62838

**Supplemental TABLES**

**Table S1 Na^+^ currents density and gating properties in tsa201 and hiPSC-CMs**

| Cells types | *I*_Na_ density  (pA/pF) | SS-inactivation | | Activation | | Recovery from inactivation | |
| --- | --- | --- | --- | --- | --- | --- | --- |
|  |  | V_1/2_ (mV) | κ (mV) | V_1/2_ (mV) | κ (mV) | τ _fast_ (ms) | τ _slow_ (ms) |
| WT-*SCN5A* tsa201 | 261.5±42.7  (n=15) | -78.52±0.33  (n=9) | 8.03±0.29 | -37.68 ±0.23  (n=9) | 4.64±0.19 | UD | UD |
| A226V-*SCN5A* tsa201 | 122.1±32.3†  (n=15) | -78.68±0.47  (n=9) | 7.51±0.40 | -34.47±0.39*  (n=9) | 5.54±0.32 | UD | UD |
| R1629X-*SCN5A* tsa201 | 4.8±2.2‡  (n=15) | UD | UD | UD | UD | UD | UD |
| CON1  (hiPSC-CMs) | 245.8±32.7  (n=15) | -70.26 ± 0.40  (n=15) | 7.55 ± 0.35 | -35.72 ± 0.46  (n=15) | 4.94 ± 0.40 | 19.1 ± 0.05  (n=14) | 327.4± 0.05 |
| BrS1  (hiPSC-CMs) | 59.2±8.8\|\|  (n=21) | -73.37 ± 0.22*  (n=15) | 6.38 ± 0.19* | -40.89 ± 0.56† (n=21) | 4.99 ± 0.49 | 23.3 ± 0.04‡  (n=14) | 380.0 ± 0.05§ |

V_1/2_: half (in)activation voltage. К: slope factor of voltage dependence of (in)activation. SS- steady state. τ _fast,_ τ _slow_: the fast and slow time constant of recovery from inactivation. * *p* <0.05; † *p* <0.01; ‡ *p* <0.001; § *p* <0.0001; || *p* <0.00001; vs. WT-*SCN5A* or Control hiPSC-CMs. Data presented are mean ± SEM. Statistics was performed with unpaired Student *t*-test (2-tails).

| *I*_Na_ density (pA/pF) | Line 1* | Line 2 | Line 3 | Line 4 |
| --- | --- | --- | --- | --- |
| CON1 (hiPSC-CMs) | 245.8±32.7 (n=15) | 227.4±29.2 (n=3) | 260.1±42.2 (n=3) | 235.4±37.8 (n=3) |
| BrS1 (hiPSC-CMs) | 59.2±8.8 (n=21) | 48.3±13.7 (n=4) | 69.9±16.7 (n=4) | 72.3±18.5 (n=3) |

* Selected for this study. A total of four lines were generated from patients and siblings. The patient lines showed consistently dramatic reduction in overall sodium current relative to unaffected sibling controls. hiPSC-CMs are notable for their variability and sensitivity to culture conditions. Thus, the high variability seen in the small samples from Line #2, #3 and #4 may not fully characterize the heterogeneity in I_Na_ current in the cells and may obscure quantitative aspects of the differences in I_Na_ between CON1 and BrS1.

**Table S2 Action potential parameters of ventricular-like hiPSC-CMs (-*I*_K1_)**

|  | Spontaneous APs | | Paced APs (BrS1 only, two batches of cells tested) | | | | |
| --- | --- | --- | --- | --- | --- | --- | --- |
|  | CON1 (n = 11)  HR: 37.83±5.81 | BrS1 (n = 11)  HR: 32.57±7.92 | 1.0 Hz (n=4) | 1.5Hz (n=3) | 1.0 Hz | 0.5 Hz | 0.2 Hz |
| APA (mV) | 87.51±5.76 | 91.16±8.09 | 71.26±1.75 | 74.12±0.93 | 73.30±1.53 | 69.00±1.55 | 64.10±5.35 |
| dV/dt_Max_ (V/s) | 17.76±4.59 | 15.76±4.59 | 5.45±0.89 | 9.21±0.09 | 10.72±0.32 | 14.05±1.87 | 13.21±0.02 |
| MDP (mV) | -43.39±2.81 | -49.09±6.12 | -57.39±1.48 | -57.87±1.54 | -57.83±0.32 | -60.72±2.02 | -53.52±3.17 |
| APD20 (ms) | 204.09±44.01 | 277.46±48.50 | 92.80±3.73 | 88.61±1.82 | 92.08±5.18 | 95.00±4.82 | 87.37±7.43 |
| APD50 (ms) | 362.09±56.35 | 430.82±60.84 | 167.94±3.84 | 153.60±5.87 | 166.10±4.77 | 164.66±9.20 | 166.30±6.97 |
| APD90 (ms) | 473.64±63.97 | 493.18±62.00 | 222.62±6.22 | 209.77±9.63 | 221.24±8.58 | 216.36±12.10 | 221.25±10.31 |
| APD20/APD90 | 0.424±0.05 | 0.563±0.07* | 0.417±0.02 | 0.42±0.01 | 0.42±0.02 | 0.44±0.01 | 0.40±0.03 |

APA: action potential amplitude. APD20, APD50 and APD90: action potential duration at 20%, 50% and 90% of repolarization. dV/dt_Max_: the maximum rate of depolarization. HR: heart rate. * *p* < 0.05; vs. control. Data presented are mean ± SEM. AP parameters recorded in spontaneously contracting control and BrS hiPSC-CMs were compared by unpaired Student *t*-test. The AP parameters of BrS paced at different frequencies were compared by two-way repeated measures ANOVA followed by the Bonferroni post hoc testing.

**Table S3 Basal AP parameters of CON1 (+*I*_k1_) and BrS1 (+*I*_k1_)**

| **1 Hz** | **CON1 (n = 22)** | **BrS1 (n = 45)** |
| --- | --- | --- |
| APA (mV) | 128.6 ± 1.3 | 115.6 ± 0.9\|\| |
| Over shoot (mV) Phase 2 | 44.4 ± 1.0 | 31.3 ± 0.9\|\| |
| RMP (mV) | -84.2 ± 0.4 | -84.4 ± 0.2 |
| dV/dt_Max_ (V/s) | 185.1 ± 11.0 | 41.9 ± 5.3\|\| |
| APD20 (ms) | 126.3 ± 14.2 | 223.8 ± 12.3\|\| |
| APD50 (ms) | 297.2 ± 25.0 | 318.2 ± 13.9 |
| APD90 (ms) | 339.2 ± 26.8 | 338.0 ± 14.3 |
| APD20/APD90 | 0.366 ± 0.02 | 0.634 ± 0.02\|\| |
| APD50/APD90 | 0.873 ± 0.02 | 0.936 ± 0.00§ |

APA: action potential amplitude. APD20, APD50 and APD90: action potential duration at 20%, 50% and 90% of repolarization. RMP: resting membrane potential. dV/dt_Max_: maximum upstroke velocity. Data presented are mean ± SEM. § *p* < 0.0001; || *p*<0.00001; vs. Control. Statistics was performed with unpaired Student *t*-test (2-tails).

**Table S4 Subgroups of BrS1 determined by changes in APD90 at different pacing frequencies**

|  | No. of cells with ratios of APD90@0.1 Hz / APD90@1.0Hz | |
| --- | --- | --- |
| Control (n=19) | n = 8 (Ratio ≥1.0) | n = 11 (Ratio 0.67~1.0) |
| BrS (n=44) | n = 19 (Ratio ≥1.0) | n = 25 (Ratio 0.20~1.0) |
| Subgroup 1: BrS non-ER (n=33, 75%) | n = 19 (Ratio ≥1.0) | n = 14 (Ratio 0.72~1.0) |
| Subgroup 2: BrS ER (n=11, 25%)* | n = 0 (Ratio ≥1.0) | n = 11 (Ratio <0.5) |

**P* < 0.05, indicating a significant difference in APD90 among total BrS hiPSC-CMs paced at different frequencies. non-ER: non-early repolarization. ER: early repolarization.

The statistics of APD90 in Control and BrS hiPSC-CMs paced at 1.0 and 0.1 Hz was performed by one-way repeated measures ANOVA followed by the Tukey's post hoc testing.

**Table S5 HR-dependent AP changes in CON1 and BrS1(+*I*_k1_)**

|  | **Control (n = 19)** | | | **BrS (non-ER, n = 33)** | | | **BrS (ER, n = 11)** | | |
| --- | --- | --- | --- | --- | --- | --- | --- | --- | --- |
|  | 1.0 Hz | 0.5 Hz | 0.1 Hz | 1.0 Hz | 0.5 Hz | 0.1 Hz | 1.0 Hz | 0.5 Hz | 0.1 Hz |
| APA (mV) | 128.0±1.4 | 129.9±1.3 | 129.9±1.3 | 115.3±0.9‡ | 117.0±1.0‡ | 114.1±1.2‡ | 116.1±2.7‡ | 112.8±2.9‡ | 103.5±4.1‡,**‡** |
| Overshoot (mV) | 43.8±1.1 | 45.5±1.0 | 45.5±1.1 | 31.1±0.9‡ | 32.7±1.0‡ | 29.7±1.2‡ | 31.1±2.6‡ | 28.3±2.8‡ | 21.9±3.6‡,**†** |
| RMP (mV) | -84.2 ± 0.2 | -84.3 ± 0.2 | -84.4 ± 0.2 | -84.2 ± 0.4 | -84.5 ± 0.4 | -84.4 ± 0.4 | -85.0 ± 0.3 | -84.5 ± 0.4 | -81.6 ± 0.9**‡** |
| dV/dtMax (V/s) | 178.9±12.3 | 193.8±13.0 | 197.3±13.9 | 36.9±5.7‡ | 40.7±6.4‡ | 42.1±6.8‡ | 36.4±6.9‡ | 36.5±7.4‡ | 35.3±7.0‡ |
| APD20 (ms) | 142.6±18.8 | 186.0±32.6 | 133.6±46.5 | 211.7±13.4 | 308.6±22.8† | 284.2±29.8‡ | 173.8±20.4 | 148.3±37.0**†** | 31.8±8.7**‡** |
| APD50 (ms) | 302.9± 32.8 | 381.7±53.0 | 328.4±65.2 | 310.9±16.1 | 424.3±26.8 | 396.0±35.2 | 249.9±17.7 | 214.9±39.7*,**†** | 65.7±10.4‡,**‡** |
| APD90 (ms) | 348.2±31.7 | 426.3±52.6 | 367.2±65.1 | 331.5±16.6 | 444.2±27.3 | 414.6±35.8 | 267.4±17.9 | 231.8±40.2*,**†** | 87.6±11.4‡,**‡** |
| APD20 /APD90 | 0.39±0.03 | 0.41±0.03 | 0.27±0.05 | 0.62±0.02‡ | 0.68±0.02‡ | 0.65±0.03‡ | 0.63±0.05‡ | 0.55±0.07 | 0.31±0.06**‡** |

APA: action potential amplitude. APD20, APD50 and APD90: action potential duration at 20%, 50% and 90% of repolarization. RMP: resting membrane potential. dV/dt_Max_: the maximum upstroke velocity. non-ER: non-early repolarization. ER: early repolarization. Data presented are mean ± SEM. Statistics was performed with two-way repeated measures ANOVA followed by the Bonferroni post hoc testing. * *p* < 0.05; † *p* <0.01; ‡ *P* < 0.001; vs. CON1 (plain symbols) or vs. subgroup 1 BrS1 (bold symbols).

**Table S6 Effects of 4-AP on the AP parameters of BrS1 (+*I*_k1_)**

| **BrS (n = 9)** | **0.1 Hz** | **0.1 Hz + 4-AP** |
| --- | --- | --- |
| APA (mV) | 107.6 ± 4.6 | 114.7 ± 1.4 |
| Overshoot (mV) | 24.7 ± 4.0 | 31.1 ± 1.2 |
| RMP (mV) | -82.9 ± 0.9 | -83.6 ± 0.6 |
| dV/dt_Max_ (V/s) | 60.7 ± 12.4 | 52.0 ± 10.0* |
| APD20 (ms) | 53.1 ± 21.3 | 405.9 ± 28.1§ |
| APD50 (ms) | 116.2 ± 31.5 | 551.5 ± 23.1\|\| |
| APD90 (ms) | 127.5 ± 29.7 | 579.3 ± 23.7\|\| |
| APD20/APD90 | 0.32 ± 0.08 | 0.70 ± 0.05† |

APA: action potential amplitude. APD20, APD50 and APD90: action potential duration at 20%, 50% and 90% of repolarization. RMP: resting membrane potential. dV/dt_Max_: the maximum upstroke velocity. * *P* < 0.05; † *p* < 0.01; § *p* < 0.0001; || *p* < 0.00001; vs. 0.1 Hz. Data presented are mean ± SEM. Statistics was performed with paired Student *t*-test.

|  |  |  |  |  |  |  |  |
| --- | --- | --- | --- | --- | --- | --- | --- |
|  |  |  |  |  |  |  |  |
|  |  |  |  |  |  |  |  |
|  |  |  |  |  |  |  |  |

**Table S7 Effects of Flecainide on the AP parameters of BrS1 (+*I*_k1_)**

| **BrS (n = 9)** | **1.0 Hz** | **1.0 Hz + Flectional** |
| --- | --- | --- |
| APA (mV) | 115.6 ± 2.2 | 109.6 ± 2.1† |
| Overshoot (mV) | 30.8 ± 1.9 | 24.6 ± 1.8† |
| RMP (mV) | -84.8 ± 0.4 | -85.0 ± 0.3 |
| dV/dt_Max_ (V/s) | 31.4 ± 7.7 | 12.6 ± 2.7† |
| APD20 (ms) | 199.9 ± 18.7 | 104.0 ± 10.5§ |
| APD50 (ms) | 288.9 ± 19.4 | 157.4 ± 12.3\|\| |
| APD90 (ms) | 308.5 ± 19.7 | 170.9 ± 12.4\|\| |
| APD20/APD90 | 0.68 ± 0.02 | 0.55 ± 0.03† |

APA: action potential amplitude. APD20, APD50 and APD90: action potential duration at 20%, 50% and 90% of repolarization. RMP: resting membrane potential. dV/dt_Max_: the maximum upstroke velocity. † *p* < 0.01; § *p* < 0.0001; || *p* < 0.00001; vs. 1.0 Hz. Data presented are mean ± SEM. Statistics was performed with paired Student *t*-test.

**Supplemental FIGURES**

**
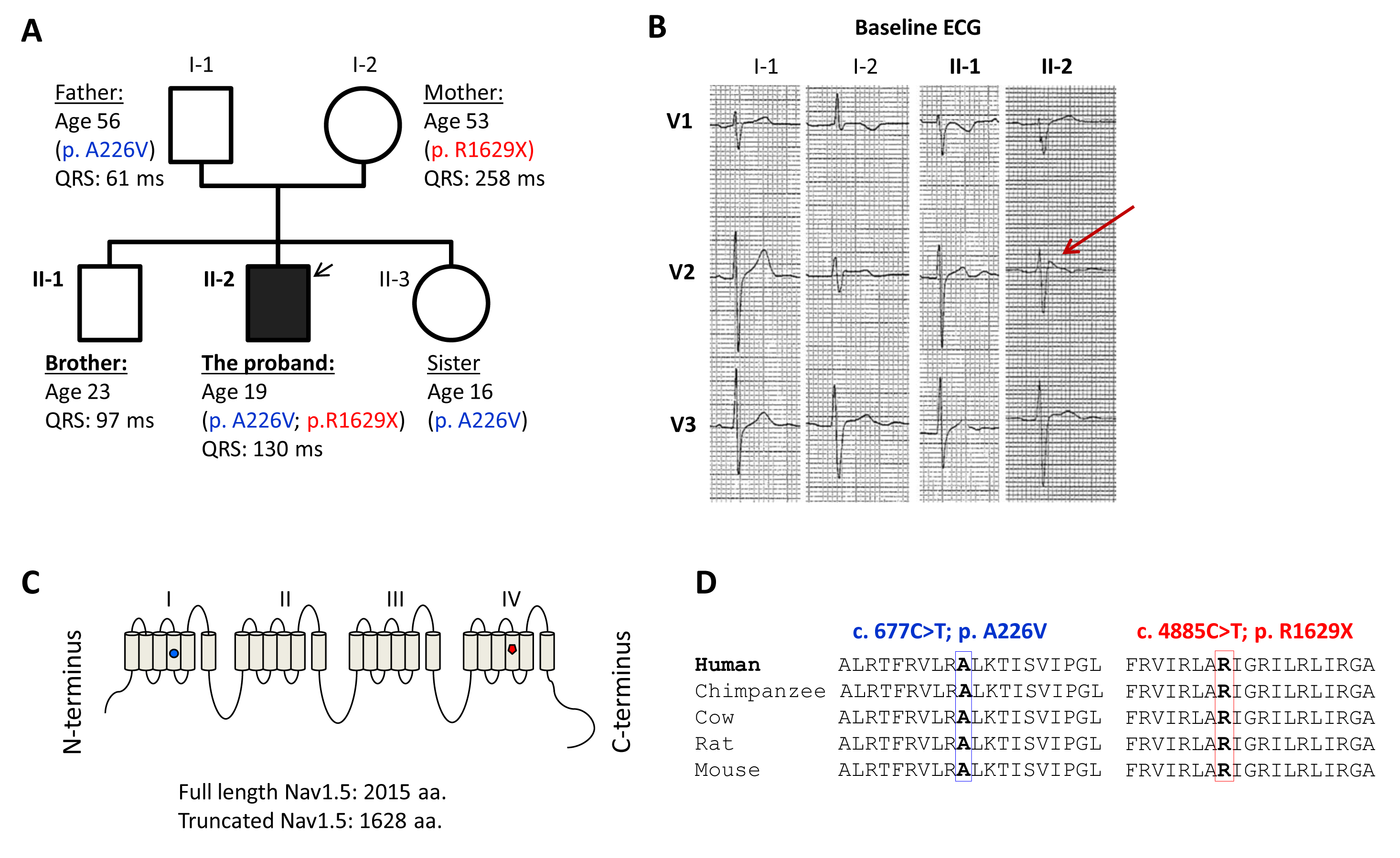
**

**Figure S1.** The BrS family. **A,** The pedigree tree of the BrS family adopted in the current study. **B**, The baseline ECG of the BrS patient and family members. **C**, The diagram of Na_v_1.5 showing the site of the SCN5A mutations.

**
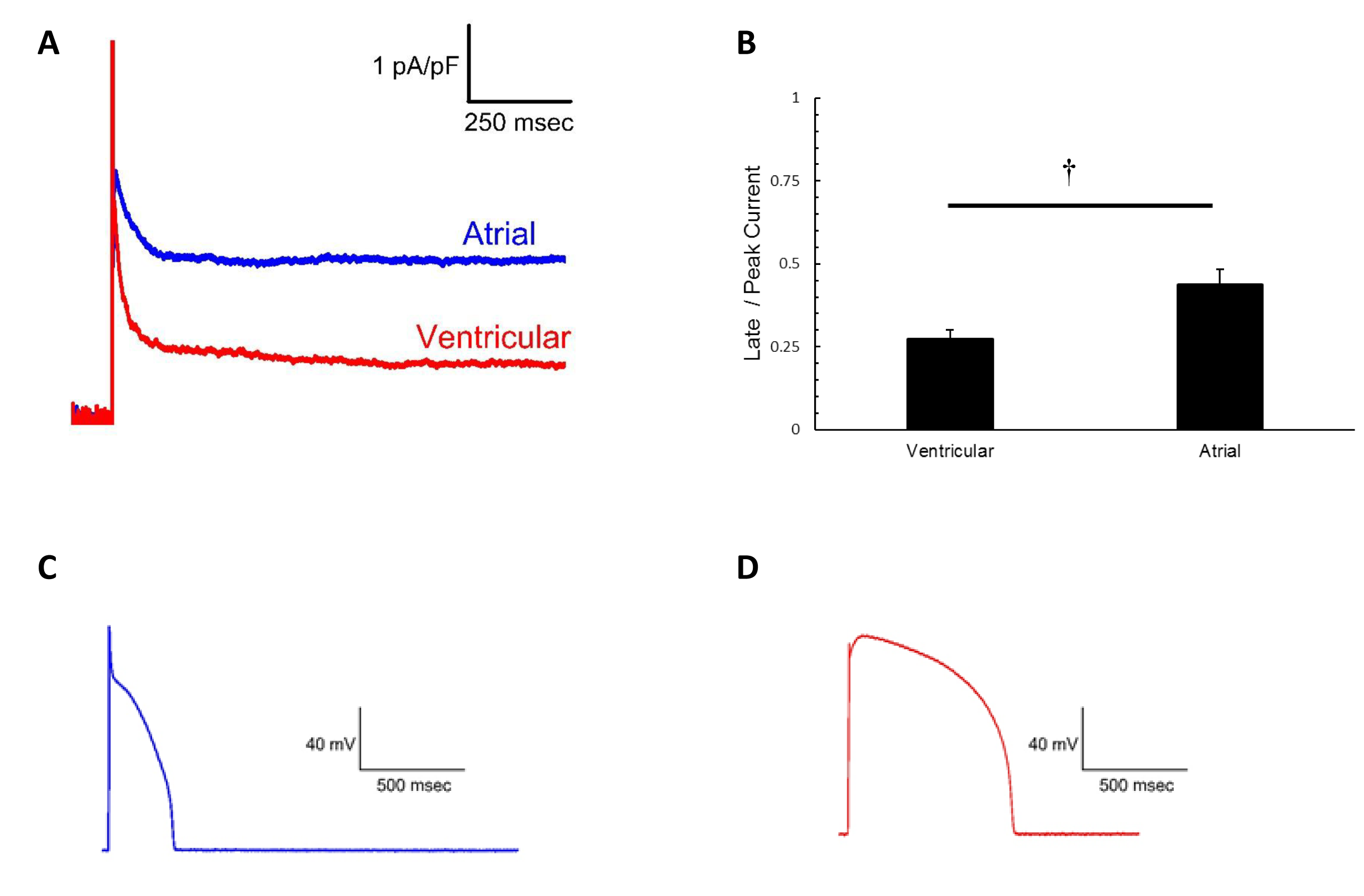
**

**Figure S2.** *I*_Kur_ currents recorded from atrial- and ventricular-like hiPSC-CMs. **A**, An example of outward K^+^ currents recorded form CON2 (iCell hiPSC-CMs) characterized as either atrial or ventricular from a holding potential of -80 mV to +50 mV. **B**, Ratio of peak to sustained late currents compared in atrial- and ventricular-like cells (Ventricular n=9, Atrial n=6). Values given are mean ± SEM. † *p*<0.01, vs. ventricular-like cells. **C** and **D**, Example atrial and ventricular action potentials based on separation by sustained peak current ratios.

**
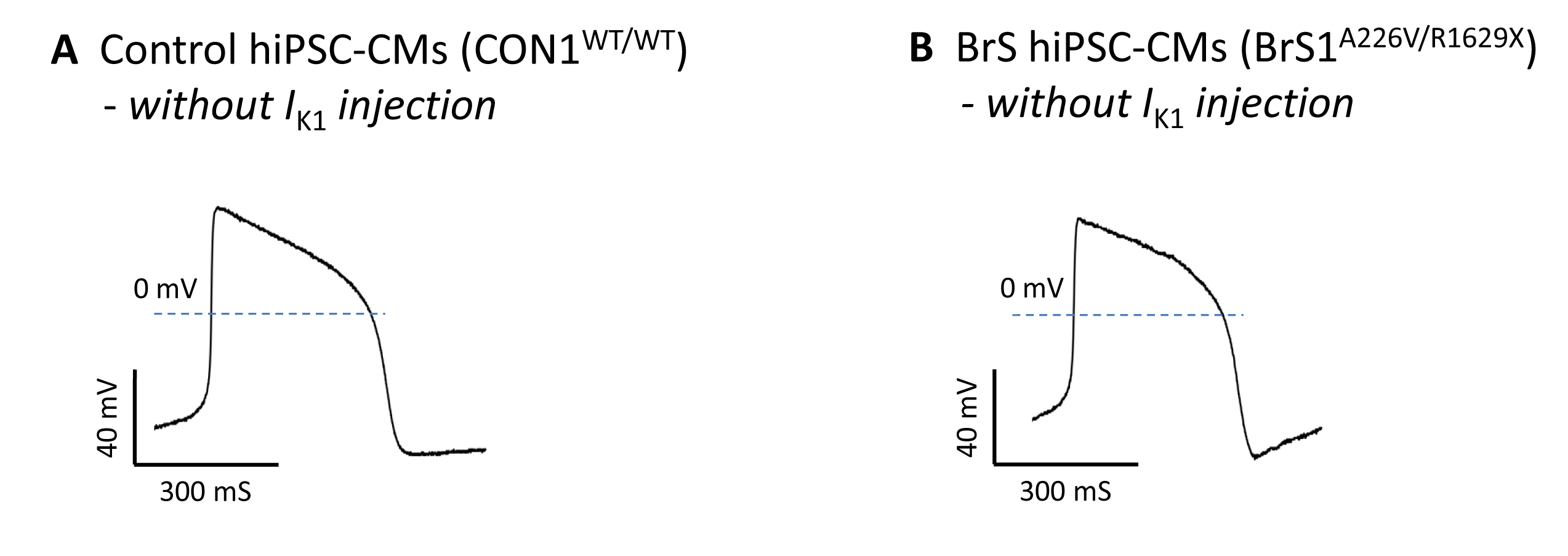
**

**Figure S3.** Action potentials of ventricular-like CON1 and BrS1 hiPSC-CMs recorded without *I*_K1_ injection. Representative AP waveforms of spontaneous contracting V-like hiPSC-CMs of CON1 (**A**) and BrS1 (**B**) recorded under conventional conditions without *I*_K1_ current injection.

**
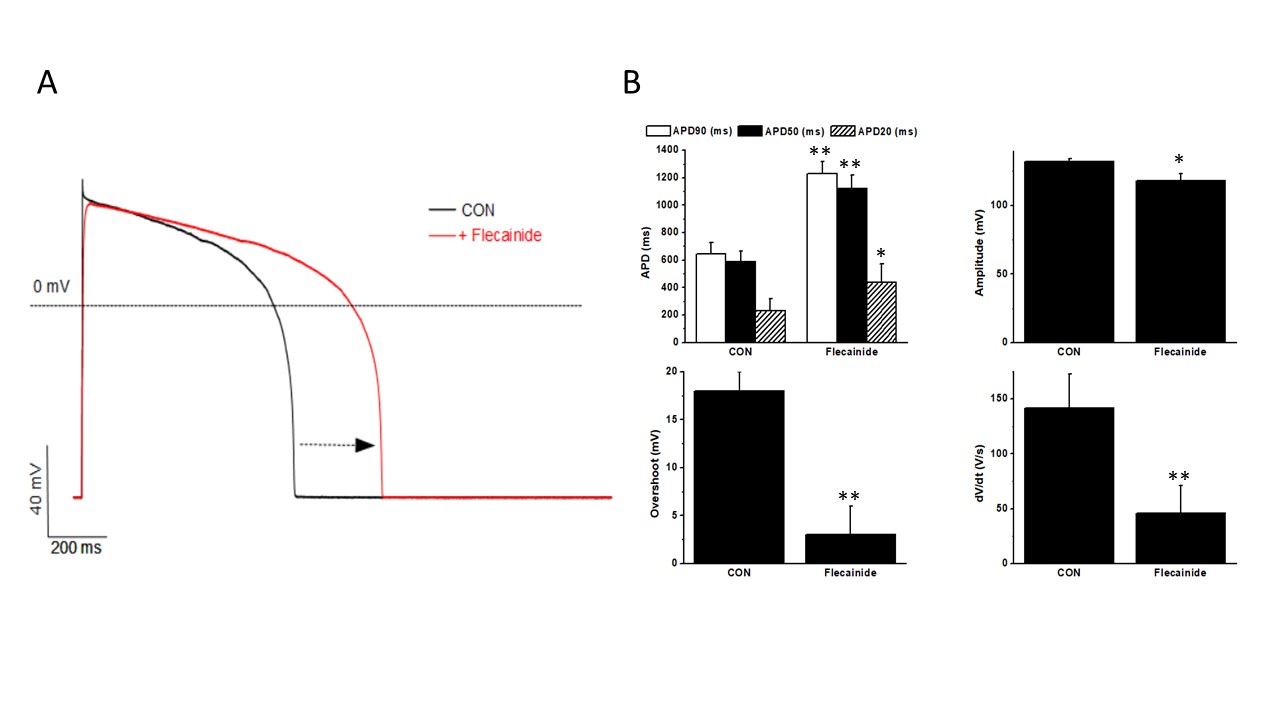
Figure S4**. Control action potential changes in response to flecainide. **A**, Representative action potential traces (0.5 Hz pacing) in the absence (black) and then in the presence (red) of flecainide (10 µM) in control hiPSC-CMs (CON2). **B**, Bar graphs showing changes in APD20, 50 and 90 as well as action potential amplitude, overshoot and dV/dt_max_ (n=4 experiments). * and ** represent p<0.05 vs CON and p<0.01 vs CON, respectively. Produced by Cytocybernetics.

**Figure S5**. Computer simulation of the effects of *I*_to_ and *I*_Ca,L_ on APDs. Adopting the O’Hara-Rudy model (2013) of human epicardial ventricular myocytes, computer simulation was performed to examine the effects of *I*_to_ and *I*_Ca,L_ on APDs. **A-left**, No APD shortening was observed in the presence of lower *I*_Na_ (*I*_Na_ reduced to 15%). **A-right**, APD shortening was observed with *I*_Na_ reduced to 15% and *I*_to_ increased to 5 folds. **B-left**, APD shortening was observed with reduced *I*_Ca,L._. **B-right**, No APD shortening was observed with reduced *I*_Ca,L_ in the presence of reduced *I*_Na_.

**
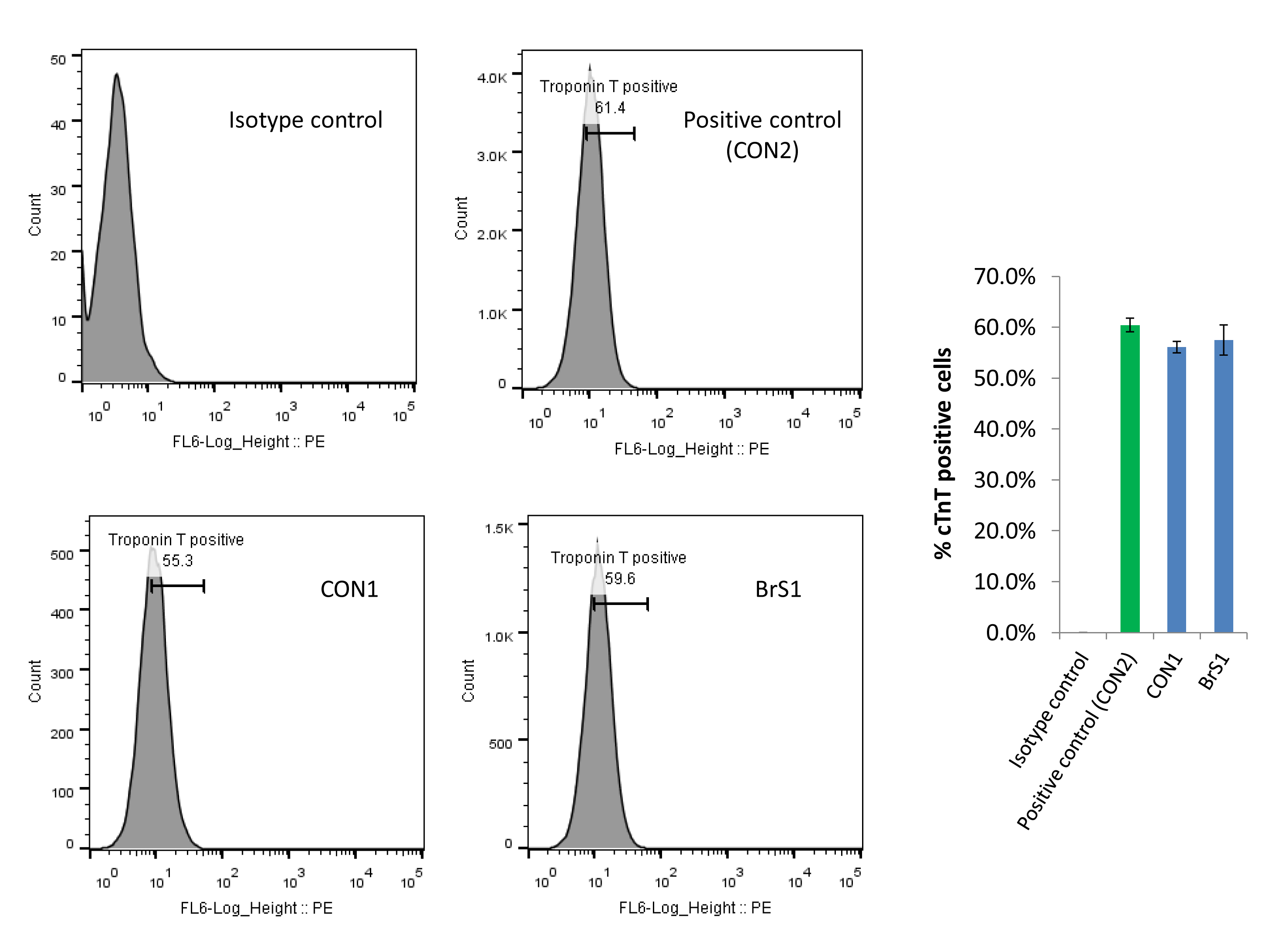
**

**Figure S6**. FACS analysis of CON1 and BrS1. CON1, BrS1 and CON2 hiPSC-CMs were stained with cTnT antibody. The experiments were repeated twice and representative diagrams of FACS were shown. Comparing with the isotype control (CON2) which was negative for cTnT, the positive control (CON2), CON1 and BrS were 60.45±1.34%, 56.10±1.13% and 57.50±2.97% positive for cTnT. Noted that the cardiac differentiation efficiency indicated by cTnT positive cells is comparable, if not better, than that in the previous studies.**^9,10^**
